# Mesenchymal stem/stromal cell-based therapy: mechanism, systemic safety and biodistribution for precision clinical applications

**DOI:** 10.1186/s12929-021-00725-7

**Published:** 2021-04-14

**Authors:** Wei-Zhan Zhuang, Yi-Heng Lin, Long-Jyun Su, Meng-Shiue Wu, Han-Yin Jeng, Huan-Cheng Chang, Yen-Hua Huang, Thai-Yen Ling

**Affiliations:** 1 Department of Biochemistry and Molecular Cell Biology, School of Medicine, College of Medicine, Taipei Medical University, 250 Wuxing Street, Taipei, 11031 Taiwan; 2 Graduate Institute of Medical Sciences, College of Medicine, Taipei Medical University, 250 Wuxing Street, Taipei, 11031 Taiwan; 3 TMU Research Center of Cell Therapy and Regeneration Medicine, Taipei Medical University, 250 Wuxing Street, Taipei, 11031 Taiwan; 4 Department of Obstetrics and Gynecology, College of Medicine, National Taiwan University, Taipei, 10041 Taiwan; 5 Department of Obstetrics and Gynecology, National Taiwan University Hospital Yunlin Branch, Yunlin, 64041 Taiwan; 6 Institute of Atomic and Molecular Sciences, Academia Sinica, Taipei, 106 Taiwan; 7 Department and Graduate Institute of Pharmacology, College of Medicine, National Taiwan University, Taipei, 10617 Taiwan; 8 Department of Chemical Engineering, National Taiwan University of Science and Technology, Taipei, 106 Taiwan; 9 International PhD Program for Cell Therapy and Regeneration Medicine, College of Medicine, Taipei Medical University, Taipei, 11031 Taiwan; 10 Center for Reproductive Medicine, Taipei Medical University Hospital, Taipei Medical University, Taipei, 11031 Taiwan; 11 Comprehensive Cancer Center of Taipei Medical University, Taipei, 11031 Taiwan; 12 The PhD Program for Translational Medicine, College of Medical Science and Technology, Taipei Medical University, Taipei, 11031 Taiwan; 13 Research Center for Developmental Biology and Regenerative Medicine, National Taiwan University, Taipei, 100 Taiwan

**Keywords:** Mesenchymal stem/stromal cell, Cell therapy, Systemic safety, biodistribution, Single cell imaging

## Abstract

Mesenchymal stem/stromal cells (MSCs) are a promising resource for cell-based therapy because of their high immunomodulation ability, tropism towards inflamed and injured tissues, and their easy access and isolation. Currently, there are more than 1200 registered MSC clinical trials globally. However, a lack of standardized methods to characterize cell safety, efficacy, and biodistribution dramatically hinders the progress of MSC utility in clinical practice. In this review, we summarize the current state of MSC-based cell therapy, focusing on the systemic safety and biodistribution of MSCs. MSC-associated risks of tumor initiation and promotion and the underlying mechanisms of these risks are discussed. In addition, MSC biodistribution methodology and the pharmacokinetics and pharmacodynamics of cell therapies are addressed. Better understanding of the systemic safety and biodistribution of MSCs will facilitate future clinical applications of precision medicine using stem cells.

**Supplementary Information:**

The online version contains supplementary material available at 10.1186/s12929-021-00725-7.

## Introduction

Cell therapy has become one of the most important emerging medical treatments in the world. Treatments utilizing stem cells, induced pluripotent stem cells (iPSCs), somatic cells, and immune cells are well documented [1]. Many cell therapy products have already received global market approval. Among them, the mesenchymal/stromal stem cells (MSCs) present a promising tool for the treatment of various diseases.

**Fig. 1:**
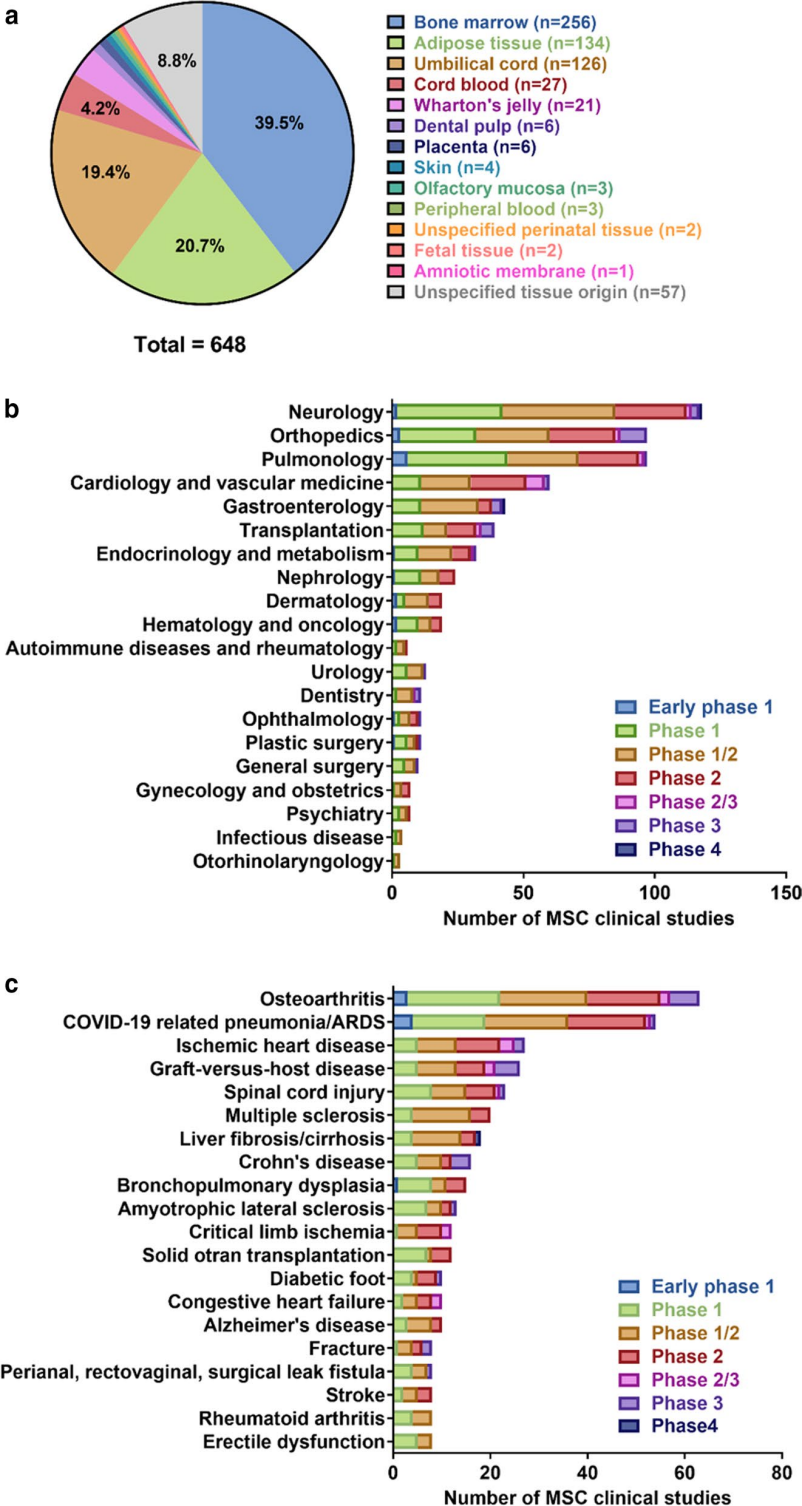
MSC sources and clinical indications in clinical studies. As of October 11, 2020, 1,242 registered studies were identified on clinicaltrials.gov by searching keywords “mesenchymal stem cell” or “mesenchymal stromal cell” (Additional file 1). After excluding studies with no longer available/ suspended/ temporarily not available/ terminated/ unknown/ withdrawn status, unknown phase information, and studies that did not use MSCs in their intervention arm, 639 studies remained. Nine of these 639 studies investigated MSCs from two tissue origins, generating a total of 648 studies for analysis. **a** Tissue origins of MSCs in clinical studies, **b** number of MSC-related clinical studies by medical specialty, and **c** the top 20 disease indications of MSC-related clinical studies

MSCs were first isolated and described by Friedenstein and his colleagues as adherent and highly replicative cells that can differentiate into mesodermal lineages including osteoblasts, chondrocytes, adipocytes, and hematopoietic stroma [2]. Since then, these cells have gained attention in the field of cell therapy for their tropism towards injured/inflamed tissues, their immunomodulatory capabilities [3], and their relative ease of isolation and expansion [4]. MSCs can be isolated from many sources, including bone marrow [5], umbilical cord [6], adipose tissue [7], cord blood [6], placenta [8], dental pulp [9], endometrium [10], amniotic fluid [11], skeletal muscle tissue [12], lung tissue [13], liver tissue [7, 12] and dermal tissue [12], and many of these cells have been used in clinical studies (Fig. 1a). The characteristics of MSCs make them attractive as cellular therapeutic agents for regenerative medicine and immune-related diseases.

The first clinical trial of MSCs was reported in 1995 in patients with hematologic malignancies. Lazarus et al. demonstrated that ex vivo expansion and subsequent infusion of human bone marrow-derived stromal progenitor cells (BMMSCs) in patients caused no severe adverse effects [14]. Subsequently, treatment with BMMSCs was shown to provide clinical improvement in the rare skeletal disease osteogenesis imperfecta [15]. Furthermore, many clinical trials have examined the feasibility and efficacy of MSCs for the treatment of various conditions, including acute organ failure [16–18], graft-versus-host disease (GVHD) [19–21], ischemic heart disease [22, 23], cardiovascular disease [24, 25], liver cirrhosis [26], diabetes [27, 28], spinal cord injury [29–31], and bone/cartilage injury [32–37] (Table 1). According to the National Institutes of Health (http://www.clinicaltrial.gov/), the number of registered MSC-based clinical trials was over 1,200 as of October 11, 2020, of which approximately 600 had defined phase and status (Fig. 1b, c, Additional file 1 and Additional file 2). Most of the studies to date are phase 1 and phase 2 trials which evaluate safety and feasibility, and evidence of therapeutic efficacy is still lacking (Fig. 1). The most common indications of MSC-based cellular therapy include osteoarthritis, ischemic heart disease, graft-versus-host disease, spinal cord injury, and multiple sclerosis (Fig. 1c). In addition, since the elevation of coronavirus disease-19 (COVID-19) outbreak to pandemic status on March 11, 2020 [38], numerous MSC-based studies have been registered, and COVID-19 related pneumonia and acute respiratory distress syndrome (ARDS) has risen as the second most common indication as of October 11, 2020 (Fig. 1c). The rapid global response and increase of COVID-19 related MSC trials highlighted the promise of MSCs in treatment of inflammatory and immune diseases.

**Table 1:** Summary of MSC-based clinical/preclinical trials

Indication	Cell source	Model	Quantification modality	In vivo distribution
Acute organ failure	Bone marrow, Bone	Rat [17, 18]	Histology/RT-PCR	More exogenous human MSCs localized to injured tissues
Graft-versus-host disease (GvHD)	Bone marrow	Patients [19]	PCR	MSC DNA detected in lymph nodes
Ischemic disease	Bone marrow	Swine [22, 23]	Histology/qPCR	DAPI staining confirmed rapid cell loss after transplantation
Lung cancer	Umbilical cord	Mouse [252]	PET-CT	MSCs remained in the lungs up to 1 week after injection
Liver cirrhosis	Bone marrow	Patients [26]	Planar whole-body acquisitions/SPECT	MSCs accumulated in the lung first, MSCs in the liver increased from 0.0%–2.8% to 13.0%–17.4% in 10 days
Diabetes	Bone marrow	Rat [28]	Histology/qPCR	MSCs detected in the diabetic kidneys at 24 and 48 h after cell infusion. Cell engraftment also observed in spleen and thymus at 24 h
Spinal cord injury	Bone marrow	Rat [299]	CT/MRI	After transplantation of BMMSCs, the hypersignal emerged in spinal cord in T1WI starting at day 7 that was focused at the injection site, which then increased and extended until day 14
Cartilage/bone injury	Adipose	Rabbit [37]	MRI	Representative tibial joint, regenerated meniscus and joint surface of tibia at 6 and 12 weeks after surgery

*BM* bone marrow, *MSCs* mesenchymal stem/stromal cells, *PCR* polymerase chain reaction, *PET* positron emission tomography, *SPECT* single-photon emission computed tomography, *MRI* magnetic resonance imaging, *CT* computed tomography

Although studies on MSCs are well-documented, MSC-based cellular products still have not been approved by the US Food and Drug Administration. The lack of consistent and standardized methods for characterizing the safety and efficacy of MSC products is a major concern, which dramatically slows the progress of MSC therapy towards clinical use. The safety of cellular products is always the first priority. Although some MSCs have been shown to be safe for clinical use in a previous meta-analysis, whether this conclusion can be extended to MSCs from other tissue origins or different culture conditions is still uncertain (Fig. 1a) [39]. The risk associated with MSC products centers around their capability to initiate and promote tumors. These risks, as well as the biodistribution of systemically administered cells must be better clarified before the widespread use of MSCs in clinical practice. In this review article, we focus on the effects of MSCs on tumor promotion and suppression, and discuss methods to study their biodistribution.

### MSC-based mechanisms of action

Several possible mechanisms by which MSCs exert their beneficial effects have been proposed. Early studies reported that MSCs could migrate to sites of injury and then differentiate into functional cells [40], or that they could fuse with compromised cells to regenerate damaged tissues [41, 42]. More recent studies have demonstrated that paracrine factors [43, 44], mitochondrial transfer [45], and extracellular vesicle secretion [46] have important roles in mediating the effects of MSCs.

### Paracrine effects

MSCs secrete paracrine factors, including cytokines, chemokines, growth factors, and miRNAs. MSC transplantation or administration of isolated secreted factors enables MSC paracrine factors to get to injured tissues, to help restore a healthy microenvironment to promote tissue repair [47] (Table 2). MSC paracrine factors play important roles in immunomodulation [48, 49], tissue regeneration and healing [50, 51], anti-fibrosis [52, 53], anti-apoptosis [54], and angiogenesis [55]. As such, many studies have focused on altering culture conditions in order to steer the secretome of MSCs towards therapeutic agents. Alterations have included using MSCs from different types of tissue [56, 57], oxygen concentration [58], growth factor incubation or cytokine pretreatment [59], passage number [60–62], three-dimensional spheroid culturing [63], and mechanical strain [64].

**Table 2:** MSC secreted factors involved in tumor promotion

Factors involved in tumor promotion		
Cytokines	IL6, TGF-β1, IL-8	[125, 132, 133, 139, 147, 150, 159, 162, 165]
Chemokines	SDF-1, CXCL1, CCL2, CCL5	[123, 124, 136, 142–144, 150, 160, 162]
Angiogenic factors	VEGF, Ang-1, PDGF, IGF	[148, 162]
Growth factor	NRG1	[135]
Other factors	periostin, PAI-1, Sema-7A	[134, 162]
microRNAs	miR-21-5p, miR-410, MiR-142-3p, miR-23b	[126, 136, 145, 158]

The capability of MSCs for immunomodulation has made them a useful treatment approach for inflammatory disorders such as multiple sclerosis [65], Crohn’s disease [66], GVHD [67], systemic lupus erythematosus [67], and type I diabetes [68]. Immunomodulation is dependent on crosstalk between MSCs and the immune microenvironment of the target tissue. In an inflammatory microenvironment, proinflammatory cytokines, including IL-1β, IL-6, IL-23, IFN-γ, and TNF-α, can stimulate MSCs to secrete anti-inflammatory factors such as TNFα stimulated gene (TSG)-6 [69], nitric oxide (NO) [70], IL-10 [71], galectins [72], prostaglandin E2 (PGE2) [73], and transforming growth factor (TGF)-β [3, 71]. Upon exposure to these MSC-secreted anti-inflammatory signals, nuclear factor (NF)-κB activity and consequent inflammatory cytokine expression in macrophages, dendritic cells, and T cells are inhibited, and immune cells will express higher levels of anti-inflammatory cytokine IL-10 as a result [3, 74]. The MSC paracrine factors also interact with other immune cells and have been reported to skew macrophage polarization towards the M2 phenotype, which downregulates both innate and adaptive immune responses [75]. Regulatory T cells (Treg) were also reported to stimulate MSCs to secrete indoleamine 2,3-dioxygenase (IDO), thereby augmenting the Treg response and attenuating acute liver injury [3, 76].

In addition to their immunomodulation ability, MSCs are able to secrete factors that can promote cell proliferation, increase angiogenesis, and reduce cell apoptosis. For example, MSCs can secrete growth and angiogenesis-promoting factors such as basic fibroblast growth factor (bFGF) [77], insulin-like growth factor (IGF) [78], TGF-β [3, 55], stromal cell-derived factor (SDF)-1α [79], secreted frizzled-related protein-1/2 (SFRP1/2) [80, 81], angiopoietins, and vascular endothelial growth factor (VEGF) [82, 83].

It has been demonstrated that MSCs can inhibit fibrosis via paracrine factors [84]. Chronic inflammation is a major factor that drives the fibrosis process, which can alter the normal architectural structure of tissues and lead to deteriorated functioning. Because MSCs can be used to reduce inflammation, they have become an attractive therapeutic strategy for suppressing fibrosis. MSC-derived conditioned medium (CM) was shown to attenuate liver fibrosis by reducing Th17 cells in a IDO-dependent manner [85]. MSC-secreted interleukin 1 receptor antagonist (IL-1Ra) was also shown to inhibit stellate cell activation and decrease type I collagen expression, a key component of liver fibrosis [86]. Administration of MSC-CM also reduced fibrotic score and collagen deposition in both bleomycin- and silica-induced lung injury models [87, 88]. In MSC-treated cells, levels of HGF, KGF, and BMP-7 increased while levels of TGF-β1 and TNF-α decreased. These results suggest that the anti-fibrotic effect of MSCs may be mediated via paracrine mechanisms [88]. In support of this, a bleomycin-induced lung injury model showed that the stanniocalcin-1 (STC-1) secreted by MSCs in response to TGF-β1 exerted antifibrotic effects by reducing oxidative stress, endoplasmic reticulum (ER) stress, and TGF-β1 production in alveolar epithelial cells [89]. Likewise, MSCs were able to decrease the expression of fibrosis-associated tissue inhibitor of matrix metalloproteinase 1 (TIMP)-1, to improve cardiac function in a myocardial infarction model [90].

### Mitochondrial transfer

Mitochondrial dysfunction is a hallmark of the aging process, and has been implicated in the pathogenesis of numerous diseases [91]. MSC-based mitochondrial transfer has therefore been a promising therapeutic strategy, by either replenishing or replacing the damaged mitochondria in targeted diseased cells [92]. Studies have observed increased tunneling nanotube (TNT) and gap junction formation with mitochondrial transfer between MSCs and injured epithelial/endothelial cells under inflammatory or hypoxic conditions, and MSC-derived mitochondria transfers could prevent apoptosis of recipient cells [93–95]. In addition, it was found that iPSC-derived MSCs could attenuate alveolar damage and fibrosis via mitochondrial transfer by TNT [96]. The tissue origin of MSCs may affect mitochondrial transfer ability. For example, iPSC-derived MSCs were shown to be more effective at mitochondria transfer compared with MSCs derived from bone marrow [96]. Mechanistically, mitochondrial transfer was found to alleviate epithelial injury through mitochondrial Rho-GTPase Miro1 regulation in an asthma model [97].

Despite these beneficial findings of MSC-mediated mitochondrial transfer, there are also potential risks, as mitochondrial transfer can increase the risk of tumor promotion. In acute myeloid leukemia (AML), NOX2 stimulated mitochondrial transfer from BMMSCs to cancer cells, and this promoted the survival of the cancer cells [98]. Mitochondrial transfer also increased the resistance of leukemic cells to chemotherapeutic agents, and transfer occurred bidirectionally [99, 100]. In an in vitro co-culture of BMMSCs and T cell acute lymphoblastic leukemia (T-ALL) cells, upon induction of oxidative stress by the addition of chemotherapeutic agents, T-ALL cells transferred their mitochondria to BMMSCs, but received few mitochondria from the BMMSCs, raising the chemoresistance of the T-ALL cells [99]. Neutralizing the cell adhesion molecule ICAM-1 and disrupting intercellular mitochondrial transfer restored the sensitivity of the T-ALL cells to the chemotherapeutic agent [99].

### Extracellular vesicle (EV) transfer

MSC-derived extracellular vesicles (EVs) have raised increasing interest as a non-cellular alternative to MSC-based therapy, as this approach eliminates concerns of unintended lineage differentiation [101]. EVs refer to exosomes, microvesicles, and apoptotic bodies, and are membrane-enclosed entities secreted by a cell in response to stimulation or apoptosis. The size and contents of these vesicles are highly variable and heterogeneous, involving proteins, mRNAs, and miRNAs [101]. Their role in MSC-mediated cellular therapy remains elusive due to their heterogeneous nature, but it is currently believed that they play an important role in many biological processes and intercellular communication [101].

Exosomes from MSCs have shown beneficial effects in disease models of autoimmune uveitis [102], retinal detachment [103], myocardial infarction [104], type 1 diabetes [105], wound healing [106], bone repair [107], burn injury [46], traumatic brain injury [108], spinal cord injury [109], and several other conditions [110]. The most commonly suggested mechanism responsible for the effects of exosomes is via their capability to regulate immune cells and immune microenvironments. MSC-derived exosomes can suppress the expression of pro-inflammatory cytokines TNF-α, IL-1β, IL-6, IL-17, IFN-γ, and MIP-1α in immune cells [103, 105, 109, 111]. Additionally, MSC-derived exosomes significantly increased the levels of anti-inflammatory cytokines IL-4, IL-10, and TGF-β in a type 1 diabetes animal model [105]. In a drug-induced liver injury model, MSC-derived exosomes enhanced the local expression of cytokines TGF-β and HGF, both of which are key factors in liver regeneration [112]. The underlying mechanism involved changes in the immune cell population, including increased M2 polarization [106, 108, 109], increased Th2 and regulatory T cell differentiation [105, 112], decreased Th17 differentiation [111], and decreased local immune cell infiltration [102].

In addition to promoting immunomodulation, MSC-derived exosomes participate in other biological processes. MSC-derived exosomes were found to promote neoangiogenesis in diabetic and burn wounds via increased VEGF-A expression, the Wnt4/β-catenin pathway, and increased tube formation and proliferation of endothelial cells [106, 113]. MSC-derived exosomes also activate Akt, ERK, and STAT3 pathways and induce expression of HGF, IGF1, NGF, SDF1, and TGF-β, which critically regulate wound healing and tissue repair [114]. In addition, MSC-derived exosomes can aid in tissue repair by enhancing autophagy and inhibiting apoptosis [103].

In contrast to microvesicles and exosomes from MSCs, apoptotic bodies are entities specifically generated by cells during apoptosis. Apoptotic bodies containing ubiquitin ligase RNF146 and miR328-3p were shown to help maintain MSC multipotency via the Wnt/β-catenin pathway [115]. In support, it was recently shown that apoptotic bodies released from donor MSCs improved myocardial infarction via autophagy regulation in recipient cells [116].

The lack of consistent or standardized methods to isolate and identify EVs presents a challenge for current therapeutics. A recent study has shown that compared to EVs, MSC-CM resulted in more effective immunomodulation [117]. Further studies are necessary to decipher the optimal MSC culture conditions and the specific subpopulations of secreted components that contribute to the most effective therapeutic benefit.

Clinical applications of MSC-derived EVs have gained increasing interest, as many of the safety concerns of MSC-based therapy might be avoided, including undesired differentiation of implanted cells in tumor formation/promotion risks, and the cell-derived secondary ischemic damage by vessel clotting. As MSC-derived EVs are still in their clinical infancy, there is currently little information on clinical safety. To monitor biodistribution, most of the in vivo studies utilize lipophilic dyes to label the EVs [118, 119]. While the injected MSC-derived EVs migrated and accumulated at the injured tissue, they also aggregated in the lung, liver, and spleen [118, 119].

### MSC safety consideration: Tumor initiation, promotion, and suppression

MSC-related cell therapy is a promising therapeutic strategy because of the high immune modulation ability and the absence of tumor initiation risk of MSCs. However, there is still concern that MSCs can pose a risk for promoting tumor cell growth [120, 121]. MSCs share some characteristics with fibroblast cells, which are able to transform into cancer-associated fibroblasts (CAFs) in tumor niches. The tumor niche involves local fibroblasts, endothelial cells, immune cells, and cancer associated MSCs. Increasing evidence shows that the tumor niche is not only trophic to cancer cells, but also highly associated with tumor initiation and growth, and is able to increase cancer stemness-related properties, including the capacity for cell migration, invasion, and chemotherapy resistance. Therefore, cancer treatment strategies have expanded from solely targeting the tumor cells, to altering the tumor milieu.

**Fig. 2:**
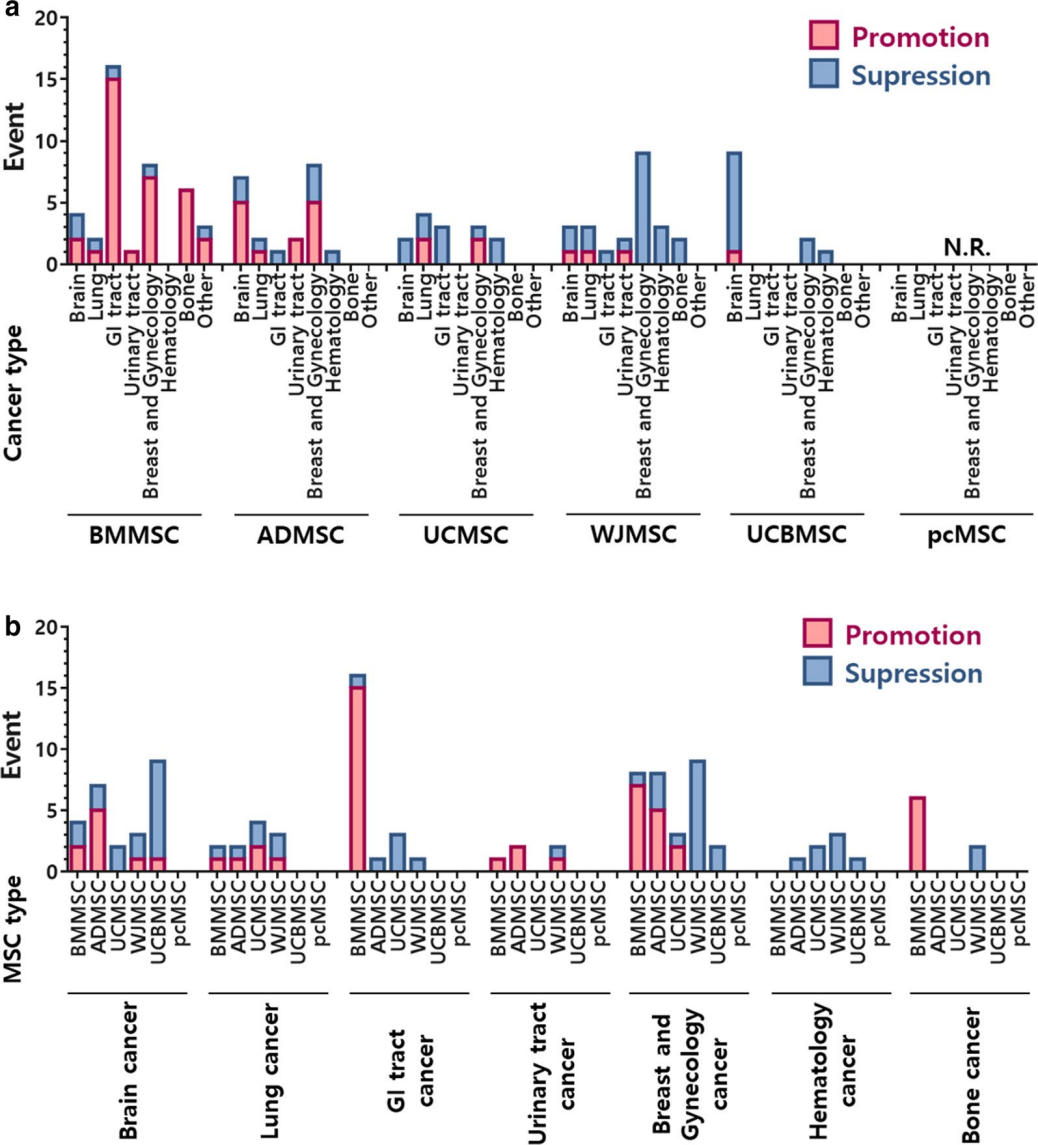
Promotion and suppression effects of MSCs on different cancer types. Data analysis from published studies listed in Tables 4 and 5, but excluding engineered MSCs. *N.R.* not reported

Since MSCs have an excellent ability for homing to tumor sites, the possibility for therapeutic MSCs to transform into cancer-associated MSCs exists. Several studies have examined the effect of MSCs on different types of tumor cells. Not surprisingly, conclusions among these studies are unclear (Fig. 2). Studies using MSCs from different tissue origins, different cultivation processes, and different cancers can lead to diverse results and interpretations.

On the other hand, taking advantage of the ability of MSCs to home to tumor sites enables MSCs to serve as therapeutic carriers that deliver anticancer agents to appropriate sites [122]. As highly progressive and late stage malignancies constitute a major health burden, for which current treatments are unsatisfactory and curative therapies are unavailable, MSC-related drug carriers may provide new hope for cancer treatments, particularly for late stage cancers.

### MSC Promotion effects on tumor cell growth and metastasis

**Fig. 3:**
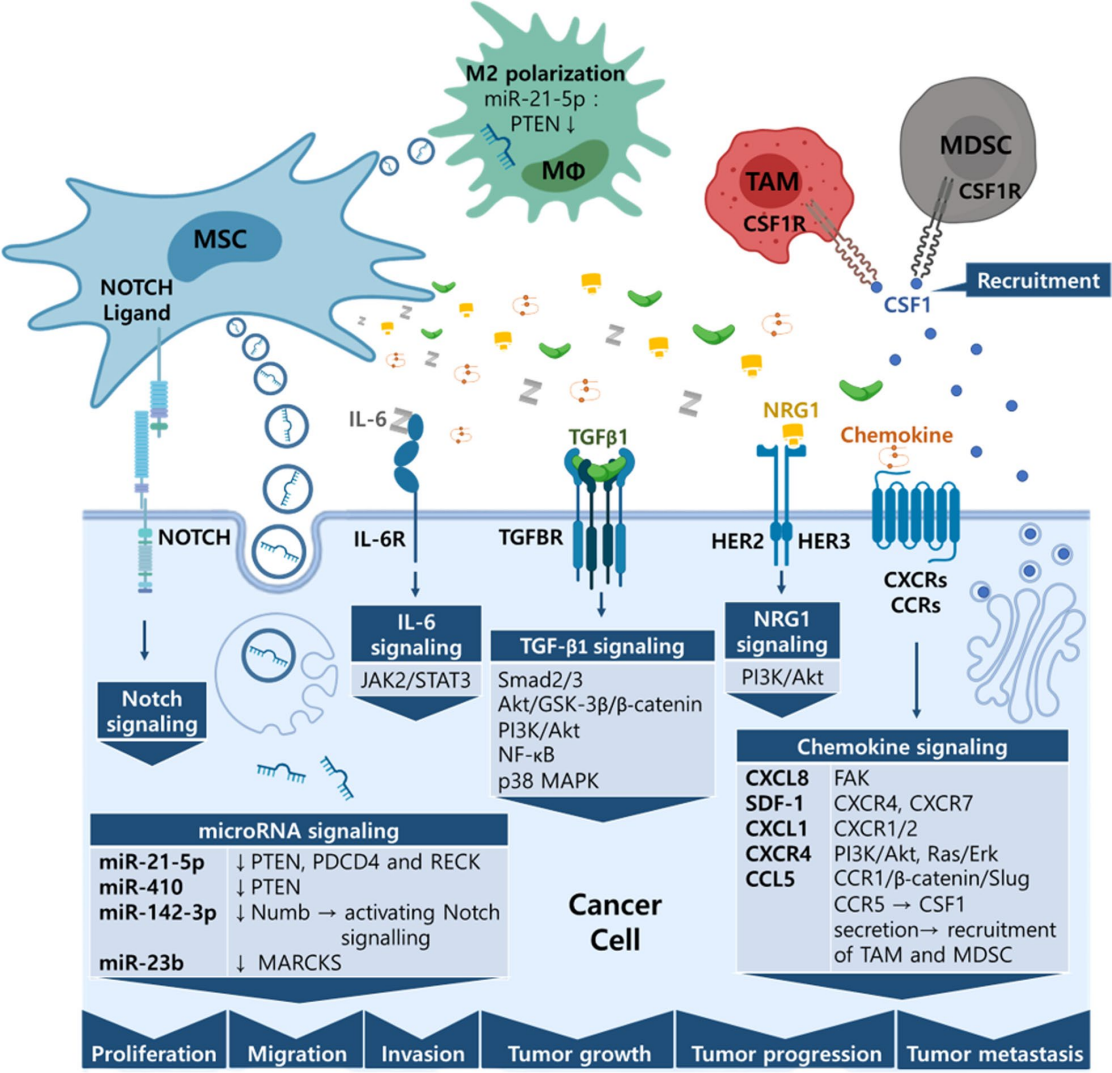
Schematic diagram of tumor promoting mechanisms of MSCs. MSCs influence cancer cells and immune cells to promote tumor cell proliferation, invasion, migration and metastasis. Secreted microRNA-containing exosomes, soluble factors, and contact-dependent signaling pathways are summarized

The underlying mechanisms responsible for MSC tumor promotion are complicated and diverse (Table 3). They are classified below according to MSC type and signaling pathway, and are listed systematically in Table 4 and summarized in Fig. 3.

**Table 3:** MSC tumor promoting signaling pathways

Tumor promoting signaling pathways		
TGF-β1 IL6 IL-8	Smad2/3, Akt/GSK-3β/β-catenin, PI3K/Akt, NF-κB, p38 MAPK JAK2/STAT3 FAK	[159] [132] [140]
SDF-1 CXCL1 CXCR4 CCL5	CXCR4, CXCR7 CXCR1/2 PI3K/Akt, Ras/Erk CCR1/β-catenin/Slug, CCR5 → CSF1 secretion → recruitment of TAM and MDSC	[124] [150] [127] [137, 142]
NRG1	HER2(HER3)/PI3K/AKT	[41]
miR-21-5p miR-410 miR-142-3p miR-23b	Downregulation of PTEN, PDCD4 and RECK; M2 polarization Downregulation of PTEN Activating Notch signalling by downregulation of Numb Downregulation of MARCKS	[126] [158] [136] [145]
Direct contact	NOTCH	[136, 144, 149]

**Table 4:** MSC promotion effects in cancers

Cancer type/MSC source	Surface marker	Effect	Factors/mechanisms	Ref.
Brain cancer				
Human **bone marrow** from ScienCell Research Laboratories	CD73^+^ CD90^+^ CD105^+^	tumor-like phenotype transformation	Glioma cell-derived exosomes, upregulating the levels of Glut-1, HK-2, and PKM-2, activating glycolysis in MSCs	[125]
Human **bone marrow** of healthy donor	SH2^+^ SH3^+^ CD29^+^ CD44^+^ CD71^+^ CD90^+^ CD106^+^ CD120a^+^ CD124^+^ CD14^−^ CD34^−^ CD45^−^	Increased tumor metastasis	SDF-1/CXCR4 and SDF-1/CXCR7 signaling	[124]
Human **adipose tissue** from trochanteric fat of healthy donor	CD73^+^ CD90^+^ CD105^+^ CD45^−^	Increased tumor cell proliferation	MSC-EV	[281]
Human **adipose tissue**	CD31^−^ CD45^−^	Increased tumor growth	Higher mRNA expression levels of angiogenic factors (VEGF, Ang-1, PDGF, and IGF) and SDF-1(CXCL12) in MSCs	[148]
Mouse **adipose tissue**	Not stated	Increased tumor migratory capacity	MSC-secreted conditioned medium increases vimentin, MMP2, and NRAS expression	[282]
Human **adipose tissue** from individuals receiving abdominoplasty	CD44^+^ CD90^+^ CD34^−^	Increased tumor growth; decreased apoptosis and H_2_O_2_-induced cancer cell death	Co-injection in vivo Co-culture or MSCs-CM	[154]
Human **adipose tissue** of individuals receiving abdominoplasty or mammoplasty	CD44^+^ CD105^+^ CD14^−^ CD34^−^ CD45^−^	Increased cancer cell migration; no significant effect on cancer cell proliferation, TMZ response, CSC traits	MSCs-CM	[282]
Human **umbilical cord Wharton's jelly** (human umbilical cord perivascular cells)	Sh2^+^ SH3^+^ Thy-1^+^ CD44^+^ CD34^−^ CD45^−^	Increased tumor growth and migration; no significant effect on TMZ response	Cytokines from MSCs-CM (ex. CCL2, PDGF-C, Sema-7A, periostin, IL6)	[162]
Human **umbilical cord blood** of healthy donors	CD29 CD44 CD73 CD90 CD105 CD166	Increased tumor growth	Exosomes CD133^+^ GBM secreted MCP-1/CCL2 and SDF-1/CXCL12 induce migration of MSCs	[160]
Lung cancer				
Human **bone marrow**	Not stated	Increased tumor growth, cancer cell proliferation, intra-tumoral angiogenesis and M2 polarization of macrophages	Downregulated PTEN, PDCD4 and RECK gene expression in cancer cells largely through miR-21-5p, which derived from EVs of MSCs pre-challenged with hypoxia	[126]
Human **adipose tissue** stem cell line	Not stated	Increased migration capacity	ADMSC-differentiated CAFs promoted cancer EMT by NOTCH pathway	[149]
Human **umbilical cord** of healthy donor	CD133^+^ CD271^+^ CD105^+^ CD3^−^ CD14^−^ CD19^−^ CD38^−^ CD66b^−^	Increased tumor EMT, invasion, and migration; decreased tumor proliferation and increased tumor apoptosis	Exosomes derived from MSCs activated Smad2/3, Akt/GSK-3β/β-catenin, NF-kB, ERK, JNK, and p38 MAPK signaling pathway by TGF-β1	[159]
Human **umbilical cord** of healthy donor	CD105 CD73 CD90 CD45 CD34 CD14 CD19 HLA-DR	Increased tumor cell proliferation and decreased tumor cell apoptosis	Reduced PTEN expression mediated by the MSC-EV-transmitted miR-410	[158]
Human **umbilical cord Wharton's jelly**	CD105^+^ CD90^+^ CD166^+^ CD73^+^ CD45^−^ CD31^−^ CD34^−^	Increased AC-LCSC tumor cell proliferation and expression of CSC markers (ALDH^+^ and CD133^+^ cell population)	MSC-CM and in vivo co-transplantation	[161]
Liver cancer				
Human **bone marrow** MSC cell line	CD44^+^ CD90^+^	Increased tumor progression, but decreased pulmonary metastasis	Decreased TGFβ1 and MMP2 expression in cancer cells	[165]
Human **bone marrow** from patients with orthopedic surgery	CD44^+^ CD73^+^ CD90^+^ CD105^+^ CD146^+^ CD34^−^ CD45^−^	Increased tumor growth, migration and invasion	MSC-dependent activation of the CXCR4, PI3K/Akt, Ras/Erk pathways	[127]
Human **bone marrow** from ATCC	Not stated	Increased tumor growth and metastasis	Activated pERK signaling pathway and over-expressed integrin α5 in HCC; decreased NK cell marker CD56 and increased IL-6 and TNF-α in tumor niche	[128]
Colorectal cancer				
Human **bone marrow** of healthy donor	CD29^+^ CD44^+^ CD73^+^ CD90^+^ CD105^+^ CD166^+^ MHC-DR^+^ CD14^−^ CD34^−^ Flk-1^−^	Increased tumor growth	MSC-differentiated CAFs expressed PDGFR to mediate tumor growth and metastasis	[130]
Human **bone marrow** of healthy donor	CD29^+^ CD44^+^ CD73^+^ CD90^+^ CD105^+^ CD166^+^ MHC-DR^+^ CD14^−^ CD34^−^ Flk-1^−^	Increased tumor growth, angiogenesis, and metastasis	MSC-differentiated into CAFs; paracrine effects of MSCs;	[129]
Human **bone marrow** of healthy donor	CD73^+^ CD90^+^ CD105^+^ CD34^−^ CD45^−^	Increased migration capacity	MSC-secreted PAI-1 promoted cancer cell migration	[134]
Human **bone marrow** of healthy donor	CD29^+^ CD44^+^ CD73^+^ CD90^+^ CD105^+^ CD166^+^	Increased tumor-initiating ability and tumor growth	MSC-secreted IL6 increased cancer cell CD133 expression by activation of JAK2/STAT3	[132]
Human **bone marrow** from sternum of healthy donor	CD49c^+^ CD73^+^ CD90^+^ CD105^+^ CD34^−^ CD45^−^ CD184^−^ CD106^−^	Increased tumor growth, invasion; decreased survival	MSC-secreted NRG1 activated HER2/HER3-dependent PI3K/AKT pathway	[135]
Human **bone marrow** from iliac crest of healthy donor	Not stated	Increased tumor growth and angiogenesis	MSC-secreted IL6 induced ET1/AKT or ERK pathway in endothelial cells to promote angiogenesis	[133]
Human **bone marrow** from the iliac crest of the patient following bone defect reconstruction	Not stated	Increased tumor growth, cell proliferation, invasion and cancer stemness-related properties	Suppressed RNA-binding protein PTBP1 by indirect co-culture with MSCs	[136]
Human **bone marrow** from the femoral head during hip-replacement surgery	Not stated	Increased cancer cells stem cell-like traits	MiR-142-3p contained in exosomes derived from MSCs promoted the Notch signalling pathway by downregulating Numb in cancer cells	[136]
Human **bone marrow** from ATCC	Not stated	Increased tumor cell proliferation, EMT, migration, and invasion	TNF-α-primed-MSCs secreted high level of CCL5 which further involved cancer cells’ CCl5/CCR1/β-catenin/Slug signaling pathway	[137]
Renal cancer				
Human **umbilical cord Wharton's jelly** of healthy donor	Not stated	Increased tumor growth	Induction of HGF synthesis via RNA transferred by MSC-MVs activated AKT and ERK1/2 signaling	[163]
Human **adipose tissue** from liposuction	CD44^+^ CD90^+^ CD34^−^ CD45^−^	Increased Ciprofloxacin resistance	Not explored	[151]
Human **amniotic fluid** from healthy pregnant women	CD44^+^ CD90^+^ CD34^−^ CD45^−^	Increased Ciprofloxacin resistance	Not explored	[151]
Ovarian cancer				
Healthy human donor	CD90 CD105 CD106 CD117 CD146 CD56 CD166 CD29 CD44 CD14 CD31 CD34 HLA-DR	Increased tumor growth and angiogenesis	LL-37 recruited MSCs, which release trophic factors that initiate angiogenesis and/or differentiate into blood vessel-supporting cells	[153]
Human **adipose tissue** from omentum of normal donors	CD105^+^ CD73^+^ CD90^+^ CD34^−^	Increased tumor growth and metastasis	Elevated the expression of MMP2 and MMP9	[152]
Endometrial cancer				
Human **adipose tissue** from omentum of patient with recurrent adenocarcinoma of endometrium and ovary	CD44^+^ CD29^+^ CD90^+^ CD105^+^ CD34^−^ CD45^−^ CD11b^−^	Increased tumor growth and cell proliferation	Not explored	[283]
Breast cancer				
Human **bone marrow** of healthy donor	Not stated	Increased tumor metastasis	MSCs involve in driving recruitment of TAMs and MDSCs	[143]
Human **bone marrow** of healthy donor	Not stated	Increased skin invasion and metastasis	Activation of EGFR signaling pathway	[284]
Human **bone marrow** of healthy donor	Not stated	Increased tumor cell migration and invasion	Tumor and stroma physical interactions activated Notch1 by TNFα or IL-1β, which further lead to CXCL8 production	[144]
Human **bone marrow** from Lonza	CD29^+^ CD44^+^ CD105^+^ CD166^+^ CD90^+^ CD73^+^ CD14^−^ CD34^−^ CD45^−^HLA-DR^−^ CD19^−^	Increased tumor lung metastases	Elevated CXCL8 (IL-8), CCL2 (MCP-1), and CCL5 (RANTES) by TNFα/IL-1β primed TNBC:MSC co-cultures	[144]
Human **bone marrow** of healthy donor	CD105^+^ CD45^−^	Increased tumor motility, invasion and metastasis	CCL5 secreted by MSCs activated CCL5-CCR5 signaling in cancer cells	[142]
Human **bone marrow** of healthy donor	Not stated	Increased tumor growth and bone metastasis	Not explored	[285]
Human **bone marrow** of healthy donor	Not stated	Acquisition of dormant phenotypes including decreased tumor cells proliferation, the abundance of stem cell–like surface markers, invasion capacity, and sensitivity to docetaxel	miR-23b contained in exosomes derived from MSCs suppressed MARCKS expression	[145]
Human **adipose tissue** from women (BMI > 30) undergoing liposuction from the subcutaneous abdominal adipose tissue	CD29 CD166 CD73 CD105 CD90 CD11b CD31 CD34 CD45 HLA-DR	Increased tumor growth and angiogenesis	CXCL1/8 secreted by MSCs activated CXCL1/8-CXCR1/2 signaling in cancer cells	[150]
Human **adipose tissue** from patients undergoing tumescent liposuction	CD44^+^ CD105^+^ HLA-ABC^+^ CD29^+^ Fik1^+^ CD45^−^ CD31^−^ CD34^−^ CD106^−^ CD184^−^	Increased tumor cell migration	Exosomes derived from MSCs activated Wnt signaling pathway	[155]
Human **adipose tissue** from ScienCell Research Laboratories (Carlsbad, CA)	CD73^+^ CD90^+^ CD105^+^	Increased Doxorubicin resistance	MSC-secreted conditioned medium promoted BCRP protein expression in cancer cells	[156]
Human **adipose tissue** from liposuction	CD73^+^ CD90^+^ CD105^+^ CD34^−^	Increased tumor metastatic spread	MSC dose dependent	[286]
Human umbilical cord of healthy donor	Not stated	Increased tumor cell invasion and metastasis	IL-8 and IL-6 secreted by MSCs activated the autocrine IL-8 and IL-6 signaling in cancer cells and induced CD44 + /CD24 − cells	[286]
Human **umbilical cord** of healthy donors	CD29^+^ CD44^+^ CD90^+^ CD34^−^ HLA-DR^−^	Increased tumor cells proliferation and migration	MSC-CM inhibited E-cadherin expression, increased the expression of N-cadherin, ZEB1 and PCNA through activation of the ERK pathway	[157]
Prostate cancer				
Mouse **bone marrow** from femurs	CD90^+^ CD34^−^/CD45^−^	Increased tumor cell migration and invasion	SDF1a secreted by MSCs	[123]
Melanoma				
Resident MSCs of bone and liver	Not stated	Increased tumor metastasis to bone and liver	MCC interact with resident MSCs at the perivascular space through co-expressed CD146 and CXCL12-CXCR4 signaling	[287]
Bone cancer				
Human **bone marrow** from the proximal femur during orthopedic surgery	Not stated	Increased tumor growth and metastasis to lung	MSC-secreted IL6 activated tumor STAT3 signaling pathway	[139]
Human **bone marrow** from patients with orthopedic surgery	CD44^+^ CD73^+^ CD90^+^ CD105^+^ CD146^+^ CD34^−^ CD45^−^	Increased tumor growth, migration and invasion	MSCs-dependent activation of the CXCR4, PI3K/Akt, Ras/Erk pathways	[127]
Human **bone marrow**	CD29^+^ CD44^+^ CD105^+^ CD45^−^ HLA-DR^−^	Increased tumor cell growth	Activation of Hedgehog signaling pathway by MSC-derived exosomes	[138]
Human **bone marrow** from TaKaRa Biotechnology	Not stated	Increased tumor cell growth and metastasis	Paracrine effect of IL-8 through activation of FAK pathway	[140]
Human **bone marrow** of healthy donor	CD73^+^ CD90^+^ CD105^+^ CD34^−^ CD45^−^ CD19^−^ CD14^−^	Increased tumor cell invasion and transendothelial migration	MSCs trans-differentiate into CAFs, increasing GRO-a, MCP-1, IL-6 and IL-8 levels in the tumor microenvironment	[141]
Human **bone marrow**	CD73^+^ CD90^+^ CD105^+^ CD34^−^ CD45^−^ CD3^−^	Increased tumor cell proliferation	Not explored	[288]
Gastric cancer				
Human **bone marrow**	CD29^+^ CD44^+^ CD105^+^ CD45^−^ HLA-DR^−^	Increased tumor cell growth	Activation of Hedgehog signaling pathway by MSC-derived exosomes	[138]
Human **bone marrow** from ATCC	Not stated	Increased tumor cells viability and invasion	MSCs recruitment and presence of CAF-like myofibroblastic phenotypes by tumor-derived HDGF	[131]
Head and neck cancer				
Human **bone marrow** from patients during hip-replacement surgery	CD73 + CD90 + CD105 + CD19- CD34-	Increased tumor cell invasion	Induction of ALP and MMP9 activity	[146]
Esophageal cancer				
Human **bone marrow**	CD29^+^ CD44^+^ CD73^+^ CD90^+^ CD45^−^ CD31^−^	Increased tumor cell proliferation, viability and invasion	Gremlin1 derived from MSC-CM is related to TGF-β/BMP signaling pathway	[147]
Bladder cancer				
Human **adipose tissue** from liposuction	CD44^+^ CD90^+^ CD34^−^ CD45^−^	Increased Ciprofloxacin resistance	Not explored	[151]
Human **amniotic fluid** from healthy pregnant women	CD44^+^ CD90^+^ CD34^−^ CD45^−^	Increased Ciprofloxacin resistance	Not explored	[151]

### Cell type

#### BMMSCs

Several studies have examined the effects of MSCs on tumor cell growth (Fig. 2). MSCs derived from human bone marrow (hBMMSCs) have been shown to enhance the motility of prostate cancer cells via SDF-1 regulation in vitro [123]. Additionally, hBMMSCs were reported to promote glioblastoma bone metastasis in vivo through the activation of SDF-1/CXCR4 and SDF-1/CXCR7 signaling [124]. It has also been shown that exosomes derived from glioma cells induce hBMMSC transformation to a tumor-like phenotype by activating glycolysis [125]. hBMMSCs that were pre-challenged with hypoxia increased tumor growth, cell proliferation, intra-tumoral angiogenesis and M2 polarization of macrophages in lung adenocarcinomas. The underlying mechanism involved downregulation of PTEN, PDCD4 and RECK gene expression by miR-21-5p derived from hBMMSCs exosomes [126]. Furthermore, hBMMSCs were shown to mediate osteosarcoma and hepatocellular carcinoma (HCC) cell migration and invasion through the regulation of CXCR4 [127]. Human MSCs (hMSCs) promote HCC tumor growth via the MAPK pathway and promote metastasis by epithelial-mesenchymal transition (EMT) and integrin α5. Furthermore, hMSC treatment promoted HCC progression, increased IL-6 and TNF-α expression, and decreased the number of natural killer (NK) cells in tumor niches [128].

In addition to their paracrine effect, hBMMSCs also promote colorectal carcinoma (CRC) and gastric cancer progression by directly differentiating to CAFs and exerting their trophic effects [129–131]. In colorectal adenocarcinomas, IL6 secreted from hBMMSCs not only increased cancer cell CD133 expression via activation of the JAK2/STAT3 pathway [132], but also activated Akt and ERK in endothelial cells by inducing the secretion of endothelin-1 (ET-1) [133]. Furthermore, hBMMSC-secreted PAI-1 and NRG1 were shown to promote CRC progression; the latter activates the PI3K/AKT pathway in a HER2/HER3-dependent manner [134, 135]. Indirect co-culture of CRCs with hBMMSCs enhanced the invasiveness of CRCs via suppression of RNA-binding protein PTBP1 [136]. The up-regulation of cancer stemness-related properties in CRCs is correlated with activation of the Notch signalling pathway by miR-142-3p, which downregulates Numb expression and is transmitted via hBMMSC exosomes[136].

One approach to mimic the inflammatory niche is to generate TNF-α-primed-hBMMSCs that secrete high levels of CCL5, which is involved in the CRC-related CCl5/CCR1/β-catenin/Slug signaling pathway that promotes tumor cell proliferation, EMT, migration, and invasion [137]. Activation of the Hedgehog signaling pathway by hBMMSC-derived exosomes leads to increased tumor cell growth in both gastric cancer and in osteosarcoma [138]. hBMMSC-secreted IL6 and IL-8 have been shown to increase tumor growth and metastasis in osteosarcomas by activation of the STAT3 and FAK signaling pathways, respectively [139, 140]. Meanwhile, elevated levels of GRO-a, MCP-1, IL-6 and IL-8 in the tumor microenvironment promoted osteosarcoma invasion and transendothelial migration via cross-talk between tumor cells and CAFs from hBMMSCs [141]. CCL5 secreted by hBMMSCs increased the motility of breast cancer cells (BCCs) by activation of CCL5-CCR5 signaling [142]. This signalling also promotes BCCs to secret CSF1, which will bind to the CSF1 receptor on MSCs, tumor-associated macrophages and myeloid-derived suppressor cells, and drive recruitment of myeloid-derived suppressor cells (MDSCs) and tumor-associated macrophage (TAMs) [143]. Elevated CCL5 (RANTES), CCL2 (MCP-1), and CXCL8 (IL-8) in TNFα/IL-1β primed triple-negative subtype of breast cancer cells (TNBCs): hBMMSCs co-cultures increase BCC lung metastases [144]. Moreover, physical interactions between TNBCs and hBMMSCs primed with TNFα or IL-1β, activates Notch1, which leads to CXCL8 production and increased tumor cell migration and invasion [144]. Exosomes derived from hBMMSCs promote the acquisition of dormant phenotypes by suppressing MARCKS expression in a bone marrow-metastatic human breast cancer cell line through miR-23b [145]. In head and neck squamous cell carcinoma (HNSCC) and esophageal squamous cell carcinoma (ESCC), increased tumor cell invasion was correlated with induction of ALP and MMP9 activity by direct contact between tumor cells and hBMMSCs, and by activation of the Gremlin1-dependent TGF-β/BMP signaling pathway by hBMMSC-CM, respectively [146, 147]

#### ADMSCs

The effect of MSCs on promoting tumor cell growth may be mediated via angiogenic factors VEGF, Ang-1, PDGF, and IGF and SDF-1 [148]. In addition, adipose tissue-derived mesenchymal stem cells (ADMSCs)-differentiated CAFs promote the EMT of lung cancers by activating the NOTCH pathway [149]. hADMSC-secreted CXCL1/8 enhances the growth and angiogenesis of BCCs by activating CXCL1/8-CXCR1/2 signaling [150]. hADMSCs and human amniotic fluid‐derived stem cells (hAFMSCs) increase ciprofloxacin resistance in renal cell carcinomas (RCCs) and bladder cancer cells [151]. Additionally, elevating the expression of MMP2 and MMP9 in ovarian cancer cells causes increased tumor growth and metastasis in both direct and indirect co-cultures with hADMSCs [152]. LL-37, which is usually overexpressed in ovarian cancer, can recruit and stimulate MSCs to release trophic factors, which increase tumor growth and angiogenesis [153]. In addition to MSCs, the CM and the EVs derived from human ADMSCs showed the ability to increase tumor growth and migration and to decrease H_2_O_2_–induced tumor cell apoptosis [154]. Meanwhile, the hADMSC-CM and exosomes were shown to increase doxorubicin resistance and tumor cell migration either by increasing breast cancer resistance protein (BCRP) levels or by activating the Wnt signaling pathway in BCCs, respectively [155, 156].

#### UCMSCs and WJMSCs

hUCMSCs promote proliferation and migration of BCCs by activating ERK signaling, including down-regulating E-cadherin expression, and up-regulating N-cadherin, ZEB1 and PCNA expression [157].

The EVs derived from hUCMSCs also have the ability to increase tumor cell proliferation and to decrease tumor cell apoptosis in lung adenocarcinomas via transmission of miR-410, which reduces PTEN expression [158]. Additionally, exosomes derived from hUCMSCs increased tumor EMT, invasion, and migration through TGF-β1-mediated signaling pathways [159]. Furthermore, CD133^+^ glioblastoma stem cells exhibited the ability to recruit hUCBMSCs, which can further promote tumor growth in vivo, via exosomes containing MCP-1/CCL2 and SDF-1/CXCL12 [160].

An increase in the cancer stemness-related ALDH^+^ and CD133^+^ cell populations was observed in lung adenocarcinomas treated with Wharton's Jelly mesenchymal stem cell CM (WJMSC-CM) [161]. WJMSC-CM also showed effects of increasing tumor growth and migration of glioblastoma cells by secreted cytokines (eg. CCL2, PDGF-C, Sema-7A, periostin, IL6) [162]. Besides the cytokines and chemokines secreted by MSCs, WJMSC microvesicles (MVs) transfer RNA to RCCs, which induces HGF synthesis and further activates AKT and ERK1/2 signaling [163].

### Signaling pathways

#### Chemokine signaling

Chemokine signaling plays an important role in MSC-dependent tumor promotion (Fig. 3). CD133^+^ glioblastoma stem cells induce hUCMSC migration to tumor regions by secreting CCL2 and CXCL12. Once in the tumor region, MSCs then promote tumor proliferation and glial invasiveness [160]. In addition, SDF-1 secreted from hBMMSCs promotes neuroblastoma migration and invasion via CXCR4 and CXCR7 [124]. hBMMSCs also enhance osteosarcoma and HCC cell migration and invasion by activating the AKT and ERK pathways of tumor cells via CXCR4 [127]. These observations suggest that chemokine signaling may be involved in bone metastasis. Furthermore, Chaturvedi et al*.* demonstrated that there is a delicate crosstalk among BCCs, hBMMSCs and TAMs/MDSCs involving chemokine signaling, and that there are two signaling loops among these cell types. In the second loop, CCL5 secreted from MSCs activates BCCs via CCR5, which promotes the BCCs to secret CSF1 and further recruits TAMs and MDSCs to the tumor region [143]. In addition, hBMMSCs weakly enhance the invasiveness and metastasis of metastatic human BCCs through CCL5-CCR5 signaling regulation [142]. CCL5 secreted from TNF-α-primed hBMMSCs also showed the ability to promote CRC progression and EMT via the CCL5/CCR1/β-catenin/Slug signaling pathway [137]. In addition to tumor and immune cells, chemokine signaling affects other cells in tumor niches. For example, CXCL1/8 derived from hADMSCs can enhance the migration and tube formation of human umbilical vein endothelial cells (HUVECs) in vitro by CXCR1 and CXCR2, which promote angiogenesis in a breast tumor xenograft mouse model [150]. CXCL8 derived from hBMMSCs was also shown to activate FAK signaling in osteosarcomas and to promote tumor metastasis [140].

#### TGF-β signaling

TGF-β is well known as an EMT promotor, but it can also induce cell cycle arrest and apoptosis [164]. In lung cancer cells, hUCMSCs have been shown to promote tumor cell EMT, invasion, and migration, but also to decrease tumor proliferation and promote tumor apoptosis by TGF-β1 from exosomes secreted by MSCs. The TGF-β1 activates Smad2/3, Akt/GSK-3β/β-catenin, NF-kB, ERK, JNK, and the p38 MAPK signaling pathway in cancer cells. Silencing TGF-β1 or inhibiting exosome secretion can eliminate the MSC-dependent effects on cancer cells described above [159]. hBMMSCs also increased tumor progression, but decreased pulmonary metastasis with decreased TGFβ1 levels in HCC [165]. Furthermore, Hong et al*.* demonstrated that hBMMSC-CM can enhance the proliferation, viability and invasiveness of esophageal cancer cells via Gremlin1, which activates the TGF-β/Smad2/3 signaling pathway by inhibiting the BMP4/Smad1/5/8 signaling pathway in cancer cells [147].

#### MicroRNA signaling

Accumulating evidence shows that EV-derived miRNA contributes to tumor initiation, angiogenesis, drug resistance, metastasis and immune suppression in cancer [166]. EVs derived from hBMMSCs pre-challenged with hypoxia can promote tumor growth, cancer cell proliferation, invasion, intra-tumoral angiogenesis and M2 polarization of macrophages in non-small cell lung cancer cells. This occurs via miR-21-5p, which decreases PTEN, PDCD4 and RECK protein levels in cancer cells while enriching for CD163^+^CD206^+^, M2 macrophage-related cell surface marker macrophages, and decreasing the CD40^+^CD86^+^, M1 macrophage-related cell surface marker macrophage population. Transfecting miR-21-5p inhibitor or re-overexpressing PTEN abrogated the tumor promoting and M2 polarization effects that the hypoxia pre-challenged EVs induced [126]. Dong et al*.* also reported that miR-410 derived from hUCMSC-secreting EVs repressed PTEN protein levels in lung adenocarcinoma cells, further increased tumor cell proliferation, and decreased tumor cell apoptosis [158].

miRNA is also reported to be involved in the dynamics of the cancer stem cell population. Increased cancer stem cell-like traits, including sphere formation, Lgr5^+^CD133^+^ population, colony formation, drug resistance, and tumourigenesis, were reported in CRCs upon treatment with hBMMSC-derived exosomes that transmitted miR-142-3p. Mechanistically, it was found that miR-142-3p inhibits the expression of the Numb gene, which results in increased mRNA and protein levels of Notch target genes Hes1, P21, and cyclin D3 mRNA [136]. On the other hand, Ono et al*.* demonstrated that miR-23b delivered via hBMMSC-derived exosomes caused bone marrow–metastatic human breast cancer cells to acquire dormant phenotypes, characterized by decreases in tumor cell proliferation, tumourigenic capacity, CD44^+^ population, invasion capacity, and sensitivity to docetaxel. The miR-23b may exert its effects by targeting MARCKS [145].

### MSC suppression effects on tumor growth

**Fig. 4:**
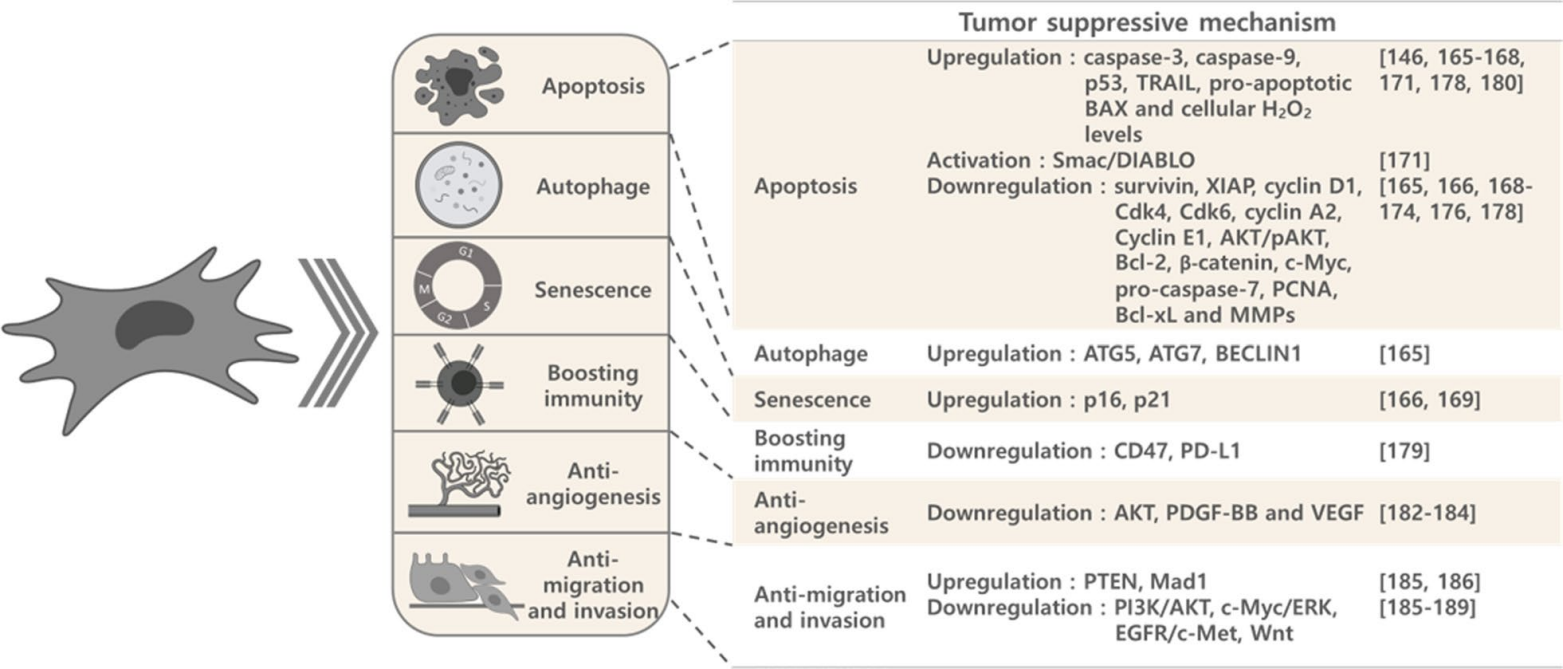
Schematic diagram of tumor suppressing mechanisms of MSCs. MSCs suppress tumor progression predominantly by promoting tumor cell apoptosis, autophagy, and senescence; and by boosting immunity, anti-angiogenesis, and anti-tumor cell migration and invasion

While MSCs utilize diverse mechanisms for tumor promotion, they suppress tumor growth mainly by inducing apoptosis of tumor cells. MSCs have been shown to suppress the growth of breast [167–169], brain [148, 170–174], lung [170, 175], liver [175, 176], ovarian [167, 177, 178], bone [167, 179], esophageal [168], bladder [180], colorectal [170] and hematological malignancies [181–183]. The underlying mechanisms responsible for MSC tumor suppression are classified below as well in Table 5**,** and are summarized in Fig. 4.

**Table 5:** MSC suppression effects in cancers

Cancer type/MSC source	Surface marker	Effect	Factors/mechanisms	Ref.
Brain cancer				
Human **bone marrow** from femoral head of individuals undergoing hip-replacement surgery	CD44^+^ CD90^+^ CD105^+^	Decreased tumor growth and angiogenesis	Coculture with hBMMSCs decreased PDGF-BB and IL1β secretion	[184]
Human **bone marrow** from Lonza	CD29^+^ CD44^+^ CD105^+^ CD166^+^ CD90^+^ CD73^+^ CD14^−^ CD34^−^ CD45^−^HLA-DR^−^ CD19^−^	Decreased tumor cell proliferation	MSC-EV	[281]
Human **bone marrow** of healthy donor	Not stated	Inducing apoptosis in CD133-positive primary glioma cells	Engineered TRAIL-expressing MSCs induced apoptosis of glioma cells	[198]
Human **bone marrow** of healthy donor	CD44^+^ CD105^+^ CD34^−^ CD38^−^ CD45^−^	Decreased cancer cell proliferation and increased animal survival	Engineered IFNβ-expressing MSCs	[201]
Human **subcutaneous adipose tissue** of 18–30 years old mothers receiving cesarean sections	CD13^+^ CD44^+^ CD90^+^ CD105^+^ CD14^−^ CD34^−^ CD45^−^	Decreased cancer cell proliferation	Increased apoptosis; G0/G1 cell cycle arrest Upregulation of caspase-3 and caspase-9 and downregulation of survivin and XIAP	[170]
Human **adipose tissue** from liposuction	CD29^+^ CD44^+^ CD90^+^ CD105^+^	Decreased tumor growth and formation	Paracrine effects by MSC-secreted cytokines	[289]
Human **adipose tissue** from patients receiving liposuctions (commercial)	CD29^+^ CD44^+^ CD73^+^ CD90^+^ CD105^+^ CD166^+^ CD14^−^ CD31^−^ CD45^−^ Lin1^−^	Decreased tumor growth, migration; induced differentiation; no significant effect in survival	Engineered BMP4-secreting MSCs had additional effect of improved survival	[204]
Human **umbilical cords** of 18–30 years old mothers receiving cesarean section	CD13^+^ CD44^+^ CD90^+^ CD105^+^ CD14^−^ CD34^−^ CD45^−^	Decreased cancer cell proliferation	Increased apoptosis; G0/G1 cell cycle arrest Upregulation of caspase-3 and caspase-9 and downregulation of survivin and XIAP	[170]
Human **umbilical cord** of healthy donor	CD73 CD105 CD90 CD45 CD34 CD14 CD11b HLA-DR	Decreased cancer stem cell proliferation	Direct cell interaction	[290]
Human **umbilical cord** from the cell library of the Chinese Academy of Science	CD29^+^ CD105^+^ CD34^−^ CD45^−^	Decreased tumor growth	Engineered IL-24-secreting MSCs induced tumor cell apoptosis	[200]
Human **umbilical cord Wharton's jelly** of healthy donor	CD13^+^ CD29^+^ CD44^+^ CD73^+^ CD90^+^ CD105^+^ CD14^−^ CD34^−^ CD45^−^ HLA-DR^−^	Decreased cancer stem-like cells proliferation	MSC-CM mediated cell cycle arrest and senescence of cancer cells by downregulating cyclin D1 and upregulating expression of p21 and p16	[171]
Human **umbilical cord Wharton's jelly** from CryoSave	Not stated	Decreased tumor cell proliferation	MSC-EV	[281]
Human **umbilical cord blood** of healthy donor	CD29^+^ CD81^+^	Decreased tumor growth and migration capacity	Co-culture with MSCs increased PTEN expression and downregulated PI3K/AKT pathway in cancer cells	[187]
Human **umbilical cord blood** of healthy donor	CD29^+^ CD81^+^	Decreased tumor growth and angiogenesis	Co-culture with MSCs downregulated FAK, VEGF, AKT	[185]
Human **umbilical cord blood** of healthy donor	CD29^+^ CD81^+^	Decreased invasion capacity	Increased cancer cell Mad1 expression represses c-Myc activity and further decreases activity of ERK	[188]
Human **umbilical cord blood** of healthy donor	CD29^+^ CD81^+^	Decreased migration and invasion capacity	Decreased EGFR and c-Met expression, activity, and physical association	[188]
Human **umbilical cord blood** of healthy donors	CD29^+^ CD81^+^	Decreased tumor growth	G0/G1 cell cycle arrest; decreased cyclin D1/Cdk4 and cyclin D1/Cdk6 expression	[172]
Human **umbilical cord blood** of healthy donors	CD29^+^ CD81^+^	Decreased tumor growth and increased apoptosis	Decreased XIAP expression resulted in activation of caspase-3 and caspase-9, downregulated AKT pathway and activated Smac/DIABLO	[173]
Human **umbilical cord blood** of healthy donors	CD31^−^ CD45^−^	Decreased tumor growth and increased tumor cell apoptosis	TRAIL signaling	[148]
Human **umbilical cord blood** of healthy donors	CD29^+^ CD44^+^ HLA-ABC^+^ CD34^−^ CD45^−^ HLA-DR^−^	Decreased tumor growth and angiogenesis	Reduced the number of cyclin D1-positive cancer cells	[174]
Human **umbilical cord blood** of healthy donors	Not stated	Decreased tumor growth and angiogenesis; increased the survival of glioma-bearing mice and tumor-specific long-term T-cell immunity	Engineered IL-12 M-secreting MSCs	[199]
Human **umbilical cord blood** of healthy donors	Not stated	Enhanced tropism of MSCs towards tumor site	Engineered CXCR4- overexpressing MSCs	[196]
Human **umbilical cord blood** from Medipost Co	Not stated	Increased tumor cell apoptosis	Engineered TRAIL-expressing MSCs induced apoptosis of glioma cells	[197]
Human **umbilical cord blood** of healthy donors	Not stated	Enhanced tropism of MSCs towards tumor site	Engineered CXCR1- overexpressing MSCs	[196]
Lung cancer				
Human **bone marrow** of healthy donor	CD29^+^ CD73^+^ CD90^+^ CD105^+^ CD166^+^ CD14^−^ CD31^−^ CD34^−^ CD45^−^	Decreased tumor tumorigenicity and EMT	MSCs-secreted OSM activates OSM/STAT1 signaling pathway	[191]
Human **subcutaneous adipose tissue** of 18–30 years old mothers receiving cesarean sections	CD13^+^ CD44^+^ CD90^+^ CD105^+^ CD14^−^ CD34^−^ CD45^−^	Decreased cancer cell proliferation	Increased apoptosis; G0/G1 cell cycle arrest Upregulation of caspase-3 and caspase-9 and downregulation of survivin and XIAP	[170]
Human **umbilical cord** of healthy donor	CD29^+^ CD44^+^ CD73^+^ CD90^+^ CD105^+^ CD14^−^ CD34^−^ CD45^−^ HLA-DR^−^	Decreased cancer cell proliferation and migration capacity	Cell cycle arrest; induced apoptosis; downregulated Bcl-2, pro-caspase-7, β-catenin and c-Myc	[175]
Human **umbilical cords** of 18–30 years old mothers receiving cesarean section	CD13^+^ CD44^+^ CD90^+^ CD105^+^ CD14^−^ CD34^−^ CD45^−^	Decreased cancer cell proliferation	Increased apoptosis; G0/G1 cell cycle arrest Upregulation of caspase-3 and caspase-9 and downregulation of survivin and XIAP	[170]
Human **umbilical cord Wharton's jelly**	CD105^+^ CD90^+^ CD166^+^ CD73^+^ CD45^−^ CD31^−^ CD34^−^	Decreased SCC-LCSC tumor cell proliferation and expression of CSC markers	MSC-CM and in vivo co-transplantation	[161]
Human **umbilical cord Wharton's jelly**	CD105^+^ CD90^+^ CD73^+^ CD45^−^ CD34^−^	No effect on AC-A549	Secretome has no effect on AC-A549	[291]
Human u**mbilical cord Wharton's jelly**	Not stated	Decreased tumor cell growth and increased apoptosis	Engineered IFNβ-expressing MSCs	[202]
Human **endometrium** of women with uterine fibroids	CD13^+^ CD29^+^ CD44^+^ CD49^+^ CD49b^+^ CD73^+^ CD90^+^ HLA-ABC^+^ CD9^−^ CD14^−^ CD31^−^ CD34^−^ CD40^−^ CD45^−^ CD54^−^ CD117^−^ CD133^−^ HLA-DR^−^	Decreased migration capacity	Not explored	[191]
Liver cancer				
Human **fetal bone marrow** at 4 months of gestation from abortion (BMMS-03)	CD105^+^ CD166^+^ CD34^−^	Decreased cancer cell proliferation	Decreased NF-κB expression and activity	[192]
Human **bone marrow** of healthy donor	CD44 + CD90 + CD34- CD45-	Decreased tumor growth	Engineered IFNβ-expressing MSCs induced cell cycle arrest and increasing expression of p21, p27 and FOXO3a as well as decreasing protein levels of cyclin D1, pRb and AKT	[203]
Human **fetal dermal tissues** at 4 months gestation from abortion (Z3)	CD105^+^ CD166^+^ CD34^−^	Decreased cancer cell proliferation	Decreased NF-κB expression and activity	[192]
Human **fetal dermal tissues** at 4 months gestation from abortion (Z3)	CD29^+^ CD44^+^ CD105^+^ CD166^+^ CD31^−^ CD34^−^ CD45^−^ HLA-DR^−^ vWF^−^	Decreased tumor growth	Induced apoptosis and cell cycle arrest; Bcl-2, c-Myc, PCNA and survivin were downregulated	[176]
Human **umbilical cord** of healthy donor	CD29^+^ CD44^+^ CD73^+^ CD90^+^ CD105^+^ CD14^−^ CD34^−^ CD45^−^ HLA-DR^−^	Decreased cancer cell proliferation and migration capacity	Cell cycle arrest; induced apoptosis; downregulated Bcl-2, pro-caspase-7, β-catenin and c-Myc	[175]
Human **umbilical cord Wharton's jelly**	Not stated	Decreased tumor growth	Engineered AFP promoter driving-sTRAIL- expressing MSCs	[208]
Bile duct cancer				
Human **umbilical cord** of healthy donors	CD29^+^ CD44^+^ CD105^+^ CD34^−^ CD45^−^	Decreased tumor growth and cell proliferation	MSC-CM inhibits Wnt/β-catenin signaling pathway by GSK-3β	[190]
Pancreatic cancer				
Human **umbilical cord blood** of healthy donors	CD44^+^ CD29^+^ HLA-I^+^ CD34^−^ CD38^−^ HLA-DR^−^	Decreased tumor growth and prolonged survival of tumor-bearing mice	Engineered IL-15- expressing MSCs mediate NK and CD8-positive T cells accumulation in tumor site	[211]
Colorectal cancer				
Human **subcutaneous adipose tissue** of 18–30 years old mothers receiving cesarean sections	CD13^+^ CD44^+^ CD90^+^ CD105^+^ CD14^−^ CD34^−^ CD45-	Decreased cancer cell proliferation	Increased apoptosis; G0/G1 cell cycle arrest Upregulation of caspase-3 and caspase-9 and downregulation of survivin and XIAP	[170]
Human a**dipose tissue** from liposuction	CD29 + CD44 + CD90 + CD105 +	Decreased tumor growth	Engineered CD-expressing MSCs sensitized colon cancer cells to 5-FC	[212]
Human **umbilical cords** of 18–30 years old mothers receiving cesarean section	CD13^+^ CD44^+^ CD90^+^ CD105^+^ CD14^−^ CD34^−^ CD45^−^	Decreased cancer cell proliferation	Increased apoptosis; G0/G1 cell cycle arrest Upregulation of caspase-3 and caspase-9 and downregulation of survivin and XIAP	[170]
Ovarian cancer				
Human **bone marrow**	CD105^+^ CD90^+^ CD44^+^ CD73^+^ CD45^−^ CD34^−^ CD24^−^ HLA-DR^−^ CD14^−^	Increased tumor cell apoptosis	MSCs-CM induced reduction in the level of CA-125, LDH and beta-hCG and expression of MMP-2, MMP-9, and CA-125 as well as increased TIMP 1, 2, and 3 mRNA expression	[177]
Human **adipose tissue** from liposuction	CD105^+^ CD90^+^ CD44^+^ CD73^+^ CD45^−^ CD34^−^ CD24^−^ HLA-DR^−^ CD14^−^	Increased tumor cell apoptosis	MSCs-CM induced reduction in the level of CA-125, LDH and beta-hCG and expression of MMP-2, MMP-9, and CA-125 as well as increased TIMP 1, 2, and 3 mRNA expression	[177]
Human **umbilical cord Wharton's jelly** of healthy donor	Not stated	Decreased tumor cell growth and migration	hWJSC-CL and hWJSC-CM induced apoptosis Upregulation of pro-apoptotic BAX and downregulation of anti-apoptotic BCL2 and SURVIVIN genes	[167]
Human **umbilical cord Wharton's jelly**	CD73^+^ CD90^+^ CD105^+^ CD34^−^ CD45^−^ CD14^−^ CD20^−^	Decreased tumor cells proliferation	MSC-CM and MSC-CL decreased the expression of oncogenic cytokines, chemokines and growth factors in cancer cells	[292]
Human **umbilical cord Wharton's jelly**	CD44^+^ CD90^+^ CD105^+^ CD73^+^ CD29^+^ CD34^−^ CD45^−^	Decreased tumor cells expression of CSC markers; increased cell cycle arrest and apoptosis	MSC-CM and MSC-CL decreased expression of cell cycle regulatory genes (cyclin A2, Cyclin E1), prostaglandin receptor signaling genes (EP2, EP4) and the pro-inflammatory genes (IL-6, TNF-α) in cancer cells	[178]
Human **umbilical cord Wharton's jelly**	Not stated	Decreased tumor cells proliferation	Not explored	[293]
Human **umbilical cord Wharton's jelly**	CD105^+^ CD90^+^ CD44^+^ CD73^+^ CD45^−^ CD34^−^ CD24^−^ HLA-DR^−^ CD14^−^	Increased tumor cell apoptosis	MSCs-CM induced reduction in the level of CA-125, LDH and beta-hCG and MMP-2, MMP-9, and CA-125 mRNA expression as well as increased TIMP 1, 2, and 3 mRNA expression	[177]
Human **umbilical cord blood** of healthy donors		Decreased tumor growth and prolonged survival of tumor-bearing mice	Engineered IL-21-secreting MSCs	[210]
Human **endometrium** of healthy women	CD73^+^ CD90^+^ CD34^−^	Decreased tumor growth and pro-angiogenetic ability	Inhibition of AKT phosphorylation and decreasing the expressions of VEGFA and HIF-1α in cancer cells, probably by activation of FoxO3a in cancer cells	[186]
Breast cancer				
Resident MSCs of vascular wall	Not stated	Decreased the risk of lung metastasis after radiation-induced injury	Downregulation of radiation-induced expression of endothelial MMP2 and of the SASP factors CCL2 and Plau/uPA	[193]
Human **adipose tissue** from normal (non-diabetic) adult lipoaspiration	CD13^+^ CD29^+^ CD44^+^ CD73^+^ CD90^+^ CD105^+^ CD166^+^ CD14^−^ CD31^−^CD45^−^	Decreased tumor cell viability and migration	MSC-secreted conditioned medium inhibits canonical Wnt signalling	[189]
Human **adipose tissue** of individuals receiving mammoplasty	Not stated	Decreased tumor cell growth and lung metastasis	Not explored	[294]
Human **umbilical cord** of healthy donors	CD73^+^ CD90^+^ CD105^+^ CD45^−^ CD14^−^	Increased tumor cells apoptosis	MSCs secretome	[295]
Human **umbilical cord Wharton's jelly** of healthy donor	Not stated	Decreased tumor cell growth and migration	hWJSC-CL and hWJSC-CM induced apoptosis Upregulation of pro-apoptotic BAX and downregulation of anti-apoptotic BCL2 and SURVIVIN genes	[167]
Human **umbilical cord Wharton's jelly** of healthy donor	CD73^+^ CD90^+^ CD105^+^ CD44^+^ CD45^−^ CD14^−^ CD34^−^	Increased tumor cells apoptosis	Upregulating p21 Downregulating PCNA, cyclin D1, Bcl-2, Bcl-xL, and MMPs and upregulating p53 and p21	[168]
Human **umbilical cord Wharton's jelly** of healthy donor	CD44^+^ CD90^+^ CD105^+^ CD34^−^	Decreased tumor cell growth	Intra-tumoral injections of MSC-CM and MSC	[179]
Mouse **umbilical cord Wharton's jelly**	Not stated	Decreased tumor cell growth	Increased activated caspase-3	[169]
Human **umbilical cord blood** of healthy donors	Not stated	Decreased tumor cell proliferation	MSC-ECM	[296]
Human **umbilical cord blood** of healthy donors	Not stated	Decreased tumor cell growth and lung metastasis	Not explored	[294]
Melanoma				
Human **bone marrow** of healthy donor	Not stated	Decreased the risk of lung metastasis after radiation-induced injury	Downregulation of radiation-induced expression of endothelial MMP2 and of the SASP factors CCL2 and Plau/uPA	[193]
Resident MSCs of vascular wall	Not stated	Decreased the risk of lung metastasis after radiation-induced injury	Downregulation of radiation-induced expression of endothelial MMP2 and of the SASP factors CCL2 and Plau/uPA	[193]
Bone cancer				
Human **umbilical cord Wharton's jelly** of healthy donor	Not stated	Decreased tumor cell growth and migration	MSC-CL and MSC-CM induced apoptosis and autophagy; upregulation of pro-apoptotic BAX and autophagy genes (ATG5, ATG7, and BECLIN1); downregulation of anti-apoptotic BCL2, SURVIVIN genes	[167]
Human **umbilical cord Wharton's jelly** of healthy donor	CD44^+^ CD90^+^ CD105^+^ CD34^−^	Decreased tumor cell growth	MSC-CL and MSC-CM induced apoptosis and autophagy	[179]
Gastric cancer				
Human **umbilical cord blood**	CD44^+^ CD105^+^ CD29^+^ CD90^+^ CD38^−^ CD117^−^ CD45^−^ CD34^−^	Increased tumor cell apoptosis	Engineered TNFSF14-secreting MSCs promote tumor cell apoptosis with elevated caspase-3 expression	[209]
Esophageal cancer				
Human **umbilical cord Wharton's jelly** of healthy donor	CD73^+^ CD90^+^ CD105^+^ CD44^+^ CD45^−^ CD14^−^ CD34^−^	Increased tumor cells apoptosis	Upregulate p21 downregulate PCNA, cyclin D1, Bcl-2, Bcl-xL, and MMPs and upregulate p53 and p21	[168]
Bladder cancer				
Human **umbilical cord Wharton's jelly** of healthy donor	Not stated	Decreased tumor growth	MSC-MVs induced down-regulated phosphorylation of Akt protein kinase and up-regulated cleaved Caspase 3	[180]
Hematological cancer				
Human **adipose tissue** from 3liposuction	CD73^+^ CD90^+^ CD105^+^ CD45^−^	Decreased tumor cell clonogenicity and growth	Not explored	[297]
Human **umbilical cord** of healthy donor	CD13^+^ CD29^+^ CD44^+^ CD73^+^ CD90^+^ CD105^+^ CD166^+^ HLA-ABC^+^ CD14^−^ CD31^−^ CD34^−^ CD38^−^ CD45^−^ HLA-DR^−^	Inducing granulocytic differentiation of APL cells	MSCs secreted IL-6 activates MEK/ERK pathways	[194]
Human **umbilical cord** of healthy donor	CD73^+^ CD90^+^ CD105^+^ CD45^−^	Decreased tumor cell clonogenicity and growth	Not explored	[297]
Human **umbilical cord** of healthy donor	CD73 CD90 CD105 CD14 CD19 CD34 CD45 and HLA-DR	Decreased tumor growth	Engineered Tandab -expressing MSCs combined with IDO pathway inhibitor inhibit expression of CD98 and Jumonji	[205]
Human **umbilical cord** of healthy donor	Not stated	Decreased tumor cell proliferation	Engineered IDO -secreting MSCs abolish the anti-apoptotic effect of MSCs	[206]
Human **umbilical cord Wharton's jelly** of healthy donor	Not stated	Decreased tumor growth and increased tumor cell apoptosis	Engineered scFvCD20-sTRAIL fusion protein -secreting MSCs	[207]
Human **umbilical cord Wharton's jelly** of healthy donor	CD105^+^ CD73^+^ CD90^+^ CD45^−^ CD34^−^ CD14^−^ CD11b^−^ CD79α^−^/ CD19^−^	Decreased tumor cells viability; increased tumor cells apoptosis	MSC-CM induced CD47 and PD-L1 expression decreased in the tumor cells	[181]
Human **umbilical cord Wharton's jelly** of healthy donor	Not stated	Decreased cell viability and mitochondrial membrane potential; increased apoptosis	MSC-CM-3 kDa MWCO regulates cellular H_2_O_2_ levels	[182]
Human **umbilical cord Wharton's jelly** of healthy donor	CD105^+^ CD73^+^ CD90^+^ CD34^−^ CD45^−^	Decreased tumor cell proliferation	IFNγ stimulated MSC secretome	[298]
Human **umbilical cord blood** of healthy donor	Not stated	Decreased tumor cell proliferation	Direct cell-to-cell contact caused arrest of the growth of cancer cells in the G0/G1 phase	[183]

### Apoptosis, autophagy and senescence

The majority of MSC tumor suppressing mechanisms involve increasing tumor cell apoptosis and impeding cell cycle progression. Upregulation of caspase-3, caspase-9, p16, p21, p53, TRAIL, pro-apoptotic BAX, ATG5, ATG7, BECLIN1 and cellular H_2_O_2_ levels [148, 167–170, 173, 180, 182]; activation of Smac/DIABLO [173]; and downregulation of survivin, XIAP, cyclin D1, Cdk4, Cdk6, cyclin A2, cyclin E1, AKT/pAKT, Bcl-2, β-catenin, c-Myc, pro-caspase-7, PCNA, Bcl-xL and MMPs have been demonstrated to be involved in the MSC-dependent tumor cell apoptosis seen with MSC-CM, MSC cell lysate (CL) and with direct cell–cell interaction [167, 168, 170–176, 178, 180].

### Boosting immunity

The immunomodulation ability of MSCs is also correlated with tumor suppression. Lin et al*.* demonstrated that 3 kDa MWCO-WJMSC-CM concentrate can induce immunogenic cell death in lymphoma cells, which showed decreased viability and increased apoptosis, as well as increased levels of the ER stress markers eLF2a and XBP-1. Increased levels of surface damage-associated molecular pattern markers ecto-CRT, ecto-Hsp70 and ecto-Hsp90, as well as extracellular ATP and high mobility group box 1 were also observed. When cocultured with WJMSC-CM-treated lymphoma cells, dendritic cells had enhanced CD80 and CD86 expression. Yet lymphoma cells treated with WJMSC-CM concentrate had decreased CD47 and PD-L1 expression [181].

### Anti-angiogenesis

In addition to directly inhibiting tumor cell growth, coculturing with hBMMSCs or hUCBMSCs decreased angiogenesis in glioblastoma. The underlying mechanism may involve the down-regulation of PDGF-BB and IL1β secretion or decreases in FAK, VEGF or Akt [184, 185]. Another attractive source of MSCs, human endometrial mesenchymal stem cells (EnSCs), also show an ability to decrease tumor growth and to increase angiogenesis in ovarian cancer by inhibiting AKT phosphorylation and decreasing expression of VEGFA and HIF-1α, possibly via nuclear translocation of FoxO3a [186].

### Anti-migration and invasion

hUCBMSCs are also reported to decrease glioblastoma cell invasion and migration by increasing PTEN or Mad1 expression and downregulating PI3K/AKT, c-Myc/ERK or EGFR/c-Met activities [187, 188]. Inhibition of Wnt signaling has been shown to decrease tumor growth and migration after treatment with hUCMSC- or hADMSC- CM in bile duct cancer and breast cancer, respectively [189, 190].

### Other mechanisms

Human BMMSC-secreted oncostatin M (OSM) has been reported to inhibit tumorigenicity and EMT by activating the OSM/STAT1 signaling pathway in lung adenocarcinoma cells [191]. Decreased cancer cell proliferation was also correlated with suppressed NF-κB expression and activity in HCCs and BCCs by MSCs derived from fetal bone marrow or fetal dermal tissue [192]. Vascular wall-resident MSCs as well as hBMMSCs displayed a capacity for decreasing the risk of lung metastasis after radiation-induced injury in breast cancer and melanoma by downregulating endothelial MMP2 and SASP factors CCL2 and Plau/uPA, which were induced by radiation injury [193]. In addition to suppressing tumor progression, hUCMSCs promote granulocytic differentiation of immature myeloid cancer cells in acute promyelocytic leukemia (APL), which drives the disease into remission by activating MEK/ERK pathways [194].

### Engineered MSCs

Another promising strategy to treat progressive malignancy is the use of engineered MSCs, which show a remarkable ability to suppress tumor progression [195]. UCBMSCs with exogenous overexpression of CXCR1 and CXCR4 displayed enhanced tropism towards gliomas [196]. In addition, irradiation of glioma cells enhanced IL-8 expression, which promoted the tropism of hUCBMSCs equipped with TRAIL migration to tumors, and further induced tumor cell apoptosis [197]. hBMMSCs overexpressing TRAIL can also induce apoptosis in CD133-positive primary glioma cells in vitro [198]. Modified interleukin-12 (IL-12p40N220Q; IL-12 M), which enhances expression of the IL-12p70 heterodimer that is necessary for induction of Th1 and CTL immunity, was overexpressed in hUCBMSCs and found to significantly decrease tumor growth and angiogenesis, as well as to increase the survival of glioma-bearing mice and to confer tumor-specific long-term T-cell immunity [199].

In human glioma studies, IL-24-hUCMSCs promoted tumor cell apoptosis, and IFN-beta-hBMMSCs were shown to prolong animal survival [200, 201]. Meanwhile, IFN-beta-WJMSCs and IFN-beta-hBMMSCs exhibited the ability to suppress tumor growth in bronchioloalveolar carcinomas [202] and HCCs, respectively, the latter exerting its effect by increasing expression of p21, p27 and FOXO3a, as well as decreasing protein levels of cyclin D1, pRb and AKT [203]. In addition, engineered BMP4-secreting hADMSCs could suppress tumor cell migratory ability and increase survival in glioblastoma [204]. As for hematological cancers, treatment with hUCMSCs equipped with Tandab (a tetravalent bispecific tandem diabody with two binding sites for CD3 and two for CD19) combined with IDO pathway inhibitor showed significantly decreased B cell lymphoma growth by way of decreasing CD98 and Jumonji, and by restoring the proliferation of T cells [205]. Another study demonstrated that UC-MSCs overexpressing IDO can inhibit proliferation of leukemia cells [206]. hWJMSCs engineered with scFvCD20-sTRAIL fusion protein, which targets CD20-positive cells and induces apoptosis through sTRAIL, inhibited proliferation in B cell lymphoma [207]. Another study showed that hWJMSCs transfected with vector coding sTRAIL driven by AFP promoter had significant antitumor activity in HCC [208]. Decreased tumor growth was also observed in gastric cancer and in epithelial ovarian cancer using hUCBMSCs delivering TNFSF14 or IL-21, respectively [209, 210]. In a syngeneic pancreatic tumor mouse model, IL15-hUCBMSCs inhibited tumor growth and increased survival of tumor-bearing mice. The IL15-hUCBMSCs induced NK- and T-cell accumulation at the tumor site and established tumor-specific T-cell memory immunity [211]. Cytosine deaminase-expressing hADMSCs serving as a prodrug converting vehicle, showed significant decreases in colorectal cancer growth in the presence of prodrug 5-fluorocytosine [212].

### Summary of promotion and suppression effects of MSCs in cancer

MSCs can contribute to tumor promotion as well as to tumor suppression. Although it may appear that these effects occur randomly, closer examination provides a more promising picture. Summarizing a total of 110 reports, (excluding engineered MSCs) reveals that in 58.6% of the studies, BMMSCs promoted tumor growth, while 9.8% of studies found that BMMSCs suppressed growth. Although the tendency of ADMSCs is not as obvious as that of BMMSCs, they also exhibit a preference for tumor promotion (Fig. 2). In general, MSCs derived from reproduction-related sources, including placenta, umbilical cord, Wharton’s jelly, and umbilical cord blood, show a higher likelihood for tumor suppression (Fig. 2). In regards to tumor type, we found that BMMSCs show an overwhelming promoting effect on cancers of the bone (100%, 6/6), breast (100%, 7/7) and GI tract, (liver, bile duct, colorectal, gastric and esophageal; 93.75%, 15/16) (Fig. 2a).

MSCs demonstrate an impressive suppressive ability in hematological cancers. In all 7 studies, MSCs from different tissue types showed tumor suppression. Similarly, in a total of 8 studies of MSCs and ovarian cancer, only one study reported that MSCs promoted tumor growth (Fig. 2b). To date, there is no report showing a tumor promoting effect for MSCs from placental tissue.

MSCs can exert their effects directly by contacting tumor cells, or indirectly by secreting soluble factors and microRNAs that the affect the tumor cells. The mechanisms by which different types of MSCs promote or suppress the growth of different tumor types are complicated (Tables 2, 3, 5, Fig. 4). Factors that may affect the properties of MSCs and cause different outcomes, include (1) the origin of the MSCs; (2) different processes of isolation, purification, and expansion of MSCs; and (3) different culture conditions and passages of the MSCs. Most of the results described herein were derived from direct or indirect in vitro co-culture systems or from in vivo co-injection experiments, but the underlying mechanisms were not always examined. It will be necessary to elucidate these underlying mechanisms, as well as to find potential biomarkers of MSC-tumor interactions for future clinical applications of MSCs.

### Biodistribution of therapeutic cells in a preclinical evaluation

In light of the tremendous potential of MSCs for treating various diseases, it is necessary to define the systemic distribution and to quantify the administered cells in order to facilitate our understanding of the safety and efficacy of MSC-based cell therapy. This information is critical in clinical trials since it is vitally important to know whether the transplanted cell products home to the target diseased sites to deliver their intended effects. Indeed, several factors can affect the pharmacokinetics (PK) of the administered MSCs, including cell size, cell source, immunological features and labeling, detection methods, route of administration, and size of the animal model.

### Factors that affect the biodistribution of MSCs

The typical diameter of a MSC is between 15–30 μm; in comparison, lymphocytes have a diameter of only 4–12 μm [213]. Furthermore, MSCs become larger after serial ex vivo cell passaging [214]. The relatively large size of MSCs explains their initial mechanical entrapment at lung capillary systems after intravenous administration, a phenomenon referred to as the pulmonary first-pass effect [26, 215]. Redistribution to liver, spleen, and other inflamed tissues subsequently takes place in the following hours to days, with gradual clearance from the lungs [26]. In some studies, MSCs were still detected in the lungs up to 150 days after transplantation in vivo [216]. MSCs retained at the lungs potentially decrease the number of cells available for therapeutic effects [217]. To decrease the mechanical entrapment of MSCs at the lungs, several strategies may be implemented, including pretreatment with the vasodilator sodium nitroprusside in order to increase the effective diameter of the pulmonary capillary system; delivery via an extravascular route; or delivery via multiple smaller doses [215, 217, 218]. Although administering MSCs intra-arterially may decrease the extent of mechanical entrapment at the lungs [219], the effect of cell size still has important implications, as larger MSCs may be associated with vascular occlusions that could cause subsequent ischemia and infarcts of unintended tissues and organs [220, 221]. Engineering of MSCs might potentially alter this adverse effect. For example, by overexpressing integrin α4 (ITGA4), which mediates leukocyte trafficking of MSCs, Cui et al. observed that cell aggregation of MSCs were significantly decreased, and MSC-associated cerebral embolism was ameliorated in rat model of stroke [222]. Furthermore, the risk of embolism has been found to be positively associated with cell dose of infusion and low infusion velocity [223].

In addition, aging of either donor or recipient could affect the biodistribution of inoculated MSCs, with decreased transplantation efficiency observed with aged donor MSCs and recipients [224]. Furthermore, when MSCs were extracted from older donors, they exhibited lower proliferative and differentiation capabilities [225, 226]. The culture condition also plays a role in the kinetics of administered MSCs. For example, hypoxic preconditioning increased MSC migration to injured tissue via enhanced HGF/cMET signaling and MSC recruitment, thus affecting biodistribution of the administered cells [227].

Immunogenic reactions also affect clearance and biodistribution of injected cells, as the allogeneic MSCs are not completely immune-privileged [228]. When MSCs are transplanted in an allogeneic host, the transplanted MSCs have decreased survival compared with their survival in a syngeneic host [229]. Formation of antibodies against injected MSCs could explain the reduced effectiveness and increased adverse effects that were observed with repeated inoculations in some studies [230].

Furthermore, the injected cells can also trigger an instant blood-mediated inflammatory reaction (IBMIR), which causes reduced graft survival and thromboembolism [231]. A portion of injected MSCs do not reach their intended destination due to the host’s immune reaction, embolization, and micro-ischemia [232]. Previous literature has demonstrated that the extent of IBMIR is related to the level of tissue factor (TF) expressed by MSCs; expression levels vary among different tissue origins of MSCs, and with culture conditions [233]. Compared with ADMSCs and UCMSCs, BMMSCs express lower levels of TF [233]. Thus, selecting TF-deficient BMMSCs may reduce the risk of IBMIR and improve the chances for clinical success. Otherwise, co-treatment with an anticoagulant may be an important consideration for clinical applications [234].

### Methods of tracking MSCs in vivo

A critical step in generating pharmacokinetic models of cell products is tracking the fate of cells following transplantation. An ideal quantification technique should have the following features: high sensitivity and specificity; long-term detection and monitoring; and spatiotemporal resolution. The advantages and disadvantages of currently available methods for quantitative MSC detection are summarized in Table 6. Polymerase chain reaction (PCR) has been used to track human MSCs in murine xenogeneic models by detecting human DNA [19, 235–237]. The low limit of detection of quantitative PCR enables detection of 100 MSCs per gram of organ tissue, making it feasible to detect MSCs in patient biopsies. Both flow cytometry and optical imaging require labeling MSCs with fluorescent dyes or proteins. Flow cytometry enables estimation of the number of live MSCs per weight unit of tissue, and optical imaging uses a variety of dyes, such as 4′,6-diamidino-2-phenlindole (DAPI), that can bind reversibly or irreversibly to the MSCs [238–241]. The use of red fluorescent protein (RFP) or green fluorescent protein (GFP) expressing MSCs has the advantage of providing viability information of transplanted cells [242]. However, the transfection efficiency is not consistent, and the transfected cells could have altered potency and expression and cannot be accurately tracked over time [243]. Therefore, the biodistribution and quantitative data produced by fluorescent protein labeling methods may be incomplete. Bioluminescence imaging (BLI) which utilizes luciferase reactions also has the advantage of providing viability information of transplanted cells, but this method suffers from poor tissue penetration and low spatial resolution. MSCs can also be labeled with gold nanoparticle and tracked by computed tomography (CT) image in vivo [244, 245]. These gold nanoparticles have advantage of exerting negligible influence on viability, proliferation, and differentiation ability of labeled MSCs, and offer good spatial resolution and long-term tracking when used in conjunction with CT modality [244]. However, sensitivity is relatively poor, and there is still difficulty deriving quantitative information from CT images [246].

**Table 6:** Comparison of methods used for quantitative mesenchymal stem/stromal cells (MSC) detection (Adapted from ref. [300])

Technique	Detection	Advantages	Disadvantages
PCR/histology	Transplanted-cell specific DNA sequences or antigens	High sensitivity No need to label the cells	Need animal sacrifice, biopsy, Postmortem samples from patients
Optical imaging	Fluorescent dyes/proteins	High throughput Good for longitudinal studies	Small animals only, Low resolution, Non-stable
Flow cytometry	Fluorescent dyes/proteins	High specificity, Quantification of live cells	Preclinical use only
MRI	Contrast agents	Clinically useful High spatial resolution Whole-body scanning	Quantification is difficult Cytotoxicity of certain labeling agents
Radionuclear	Radioisotope labels	Quantification feasible using SPECT Whole-body scanning High sensitivity	Limited spatial resolution Ionizing radiation
FND	Fluorescence	Large animal models (pigs) PK/PD of transplanted cells Biodistribution of transplanted cells Background-free imaging Single-cell detection sensitivity High throughput quantification No interference with cell potency	Need animal sacrifice

*PCR* polymerase chain reaction, *PET* positron emission tomography, *SPECT* single-photon emission computed tomography, *FND* fluorescent nanodiamond, *PK* pharmacokinetic, *PD* pharmacodynamics

Magnetic resonance imaging (MRI) can be used to track MSCs in vivo by labeling MSCs with superparamagnetic iron oxide nanoparticles (SPIONs) or fluorine-19 (^19^F). Direct labeling of MSCs with SPIONs is possible as these agents are readily taken up by MSCs and show up as hypointense signals on MRI [247]. However, some studies have shown that proliferative and differentiation capabilities of MSCs could be affected when labeled at higher concentrations [247]. The downside of SPION labeling is that the specificity of SPION-labeled cells could be low and the signals could be hard to differentiate from acutely injured tissues containing hemorrhages. In contrast, ^19^F-labeling offers better specificity as endogenous fluorine level is low, minimizing background interference and is a better labeling agent when the targeting lesion involves hemorrhage [248]. In general, MRI offers good spatial resolution but suffers from poor temporal resolution. Positron emission tomography (PET), single-photon emission computed tomography (SPECT) [26, 249–251] and radioisotope labeling [26, 252, 253] have been used to image and track the migration dynamics, and inter-patient variability of MSCs in clinical patients, but quantifying cell numbers with these methods is difficult and only semi-quantitative information on the biodistribution of the transplanted cells can be obtained. Photoacoustic imaging, which combines ultrasonography with optical imaging, is another attractive approach, as ultrasonography has the unique advantage of providing real-time information while still maintaining good spatial resolution. By using gold nanorods coated with reactive oxygen species (ROS) sensitive dye as probe, Dhada et al. were able to also detect viability of implanted cells [254]. However, photoacoustic imaging suffers from operator dependent variability [255]. More recently, multimodal imaging probes that combine the advantage of different imaging modalities have been developed, including PET/MRI imaging agent [256], SPECT/MRI/fluorescent imaging agent [257], and SPECT/MRI/BLI imaging agent [258].

An ideal cell tracking method should be biocompatible and nontoxic, require no genetic modification, have single-cell detection sensitivity, and permit quantification of cell numbers at any anatomic location. Optical imaging utilizing nanoparticles as exogenous contrast agents is suitable for this purpose, although the technique is mainly used for animal models in preclinical experimentation due to the limited penetration depth of visible photons into tissue. Among various exogenous contrast agents, fluorescent nanodiamond (FND) has emerged as an attractive option because it is chemically inert and inherently biocompatible [259, 260]. A viable application of FNDs for background-free imaging and quantitative tracking of MSCs in animal models beyond rodents has been demonstrated using magnetic modulation [261–263]. The magnetic modulation fluorescence (MMF) method uses magnets to modulate the fluorescence intensity of FNDs. This technique, which allows background-free imaging, together with the inertness of FNDs and the large quantity of the nanoparticles taken up by the cells, has permitted studies of the biodistribution and pharmacokinetics of FND-labeled MSCs in preclinical settings. This strategy can also be applied to the characterization of cell-based products in order to accelerate their progression towards commercialization to meet the needs of patients. The technique has excellent compatibility with time-gated fluorescence imaging, which has been shown to be a powerful means of acquiring high-contrast fluorescence imaging of FND-labeled cells in tissues. The ability to find single cells is particularly valuable for ex vivo histological detection of MSCs in clinical trials. This combined approach represents an appealing alternative to hazardous radioisotope labeling techniques in cell tracking applications. The technique can be used with immune cells, stem cells, and other cell types used for cell therapy. Here, we put these technologies together, and describe how they could be used to contribute to the development of pharmacokinetic modeling of MSC-based cell products.

### An FND-based platform to track therapeutic cells in vivo

**Fig. 5:**
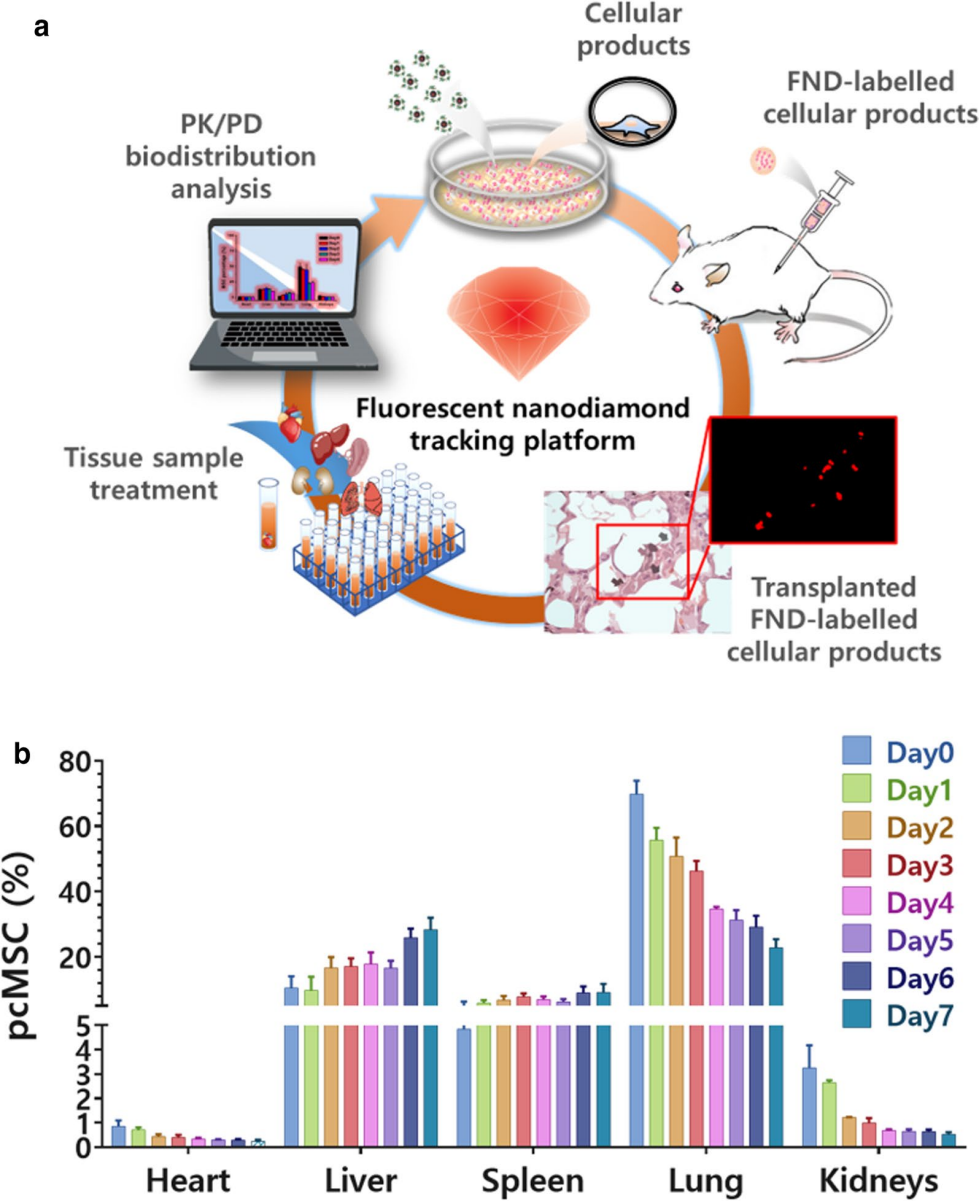
Workflow of fluorescent nanodiamond (FND)-labelled tracking platform and biodistribution analysis of FND-labelled pcMSCs. **a** The FND-labelled tracking platform for cell biodistribution analysis. This platform can provide analysis for transplanted cell localization, pharmacokinetics (PK), and pharmacodynamics (PD). FND-labelled cells are delivered through intravenous injection. The transplanted cells can be pinpointed to specific locations with background-free imaging by Leica SP8 microscopy using a time-gating technique. PK and PD analyses can be performed with a magnetic modulation fluorescence (MMF) machine after tissue/organ digestion. **b** Distribution of FND-labelled pcMSCs among different organs in a healthy mouse model. Experiments were repeated in triplicate and error bars represent the standard deviation

The ability to monitor the behavior of transplanted cells in vivo is required for cell therapy. When cellular products are submitted for investigational new drug (IND) status, pharmaceutical studies must provide evidence of not only the safety of the cell product, but also information regarding cell location, cell migration, PK and pharmacodynamics (PD), and cell biodistribution after transplantation in animal models. There are three critical issues that must be addressed for cell therapy: (1) whether therapeutic cells maintain their potency after transplantation, (2) the appropriate dosage for curing diseases and (3) a route of administration and a formulation that permits successful drug delivery. Over the past decade, the traditional concepts, confined to low molecular weight organic compounds and large biomolecules, have been challenged with the advent of new drugs based upon cells, which we refer to here as cell therapy. As for all drugs, understanding the pharmacology of cell-therapy products is critical for their effective application in the clinical setting. For example, tissue section and PCR does not provide sufficient information of cell behavior in vivo, because these procedures select a sample from a population, making it difficult to provide PK and PD information for the whole animal. In contrast, the FND-labelled tracking technique provides a new method to achieve high throughput whole organ treatment and analysis, providing accurate pharmacology information, such as PK, PD and biodistribution of the cellular therapy (Fig. 5a). This method not only provides immediate and highly specific cell localization data after gathering histological sections from the animal, but also provides a one-step, one-tube analysis for any kind of animal tissue. Compared to the qPCR sampling method, this protocol can provide more accurate data for whole organ/tissue analysis and takes less time for validation and analysis.

We use a healthy mouse model to demonstrate that the FND-labelled platform can provide evidence of cell biodistribution. Figure 5b shows the biodistribution analysis of FND-labelled placenta choriodecidual membrane-derived MSCs (pcMSCs) for one week in a mouse model using the FND-based labelling platform. Our results show that the majority (up to 70%) of FND-labelled pcMSCs localized to the lungs after intravenous administration, which is consistent with the pulmonary first-pass effect [217, 264]. The trapping of MSCs in the lungs is due to space restriction [265], as pcMSCs are more than ~ 20 μm in diameter and much larger than the width of the micro-capillaries of the lung. After intravenous infusion, FND-labelled pcMSCs disappeared from the lungs as time passed, and migrated to other tissues/organs such as the liver and spleen, or to injured sites. Nevertheless, the number of FND-labelled pcMSCs decreased in the heart and kidneys (Fig. 5b).

**Fig. 6:**
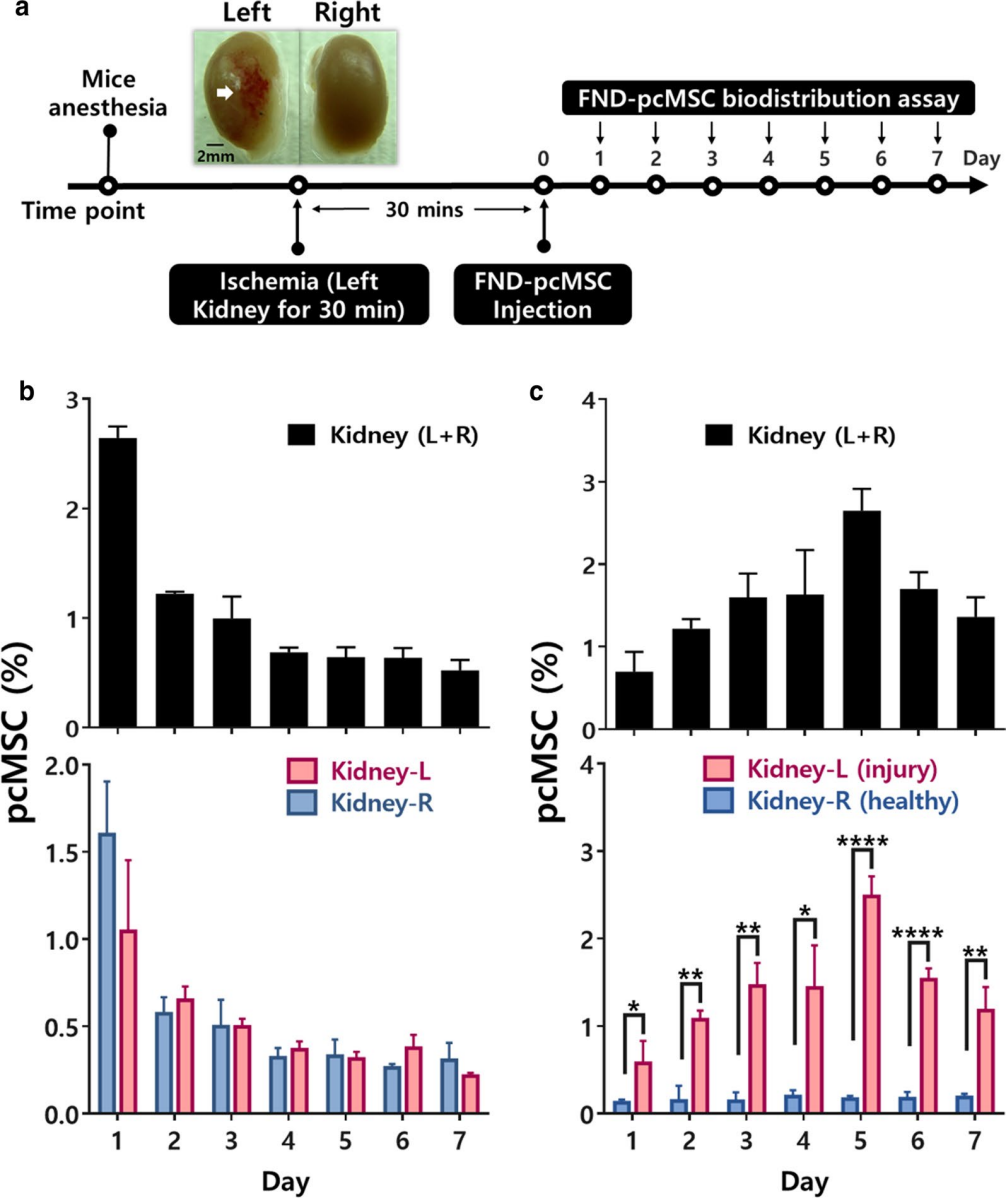
Fluorescent nanodimond (FND)-labelled pcMSC biodistribution analysis in mouse model with a kidney ischemia–reperfusion injury. **a** Timeline of the ischemia–reperfusion kidney injury mouse model. The ischemia–reperfusion injury was created on the left-hand side kidney (L) in a mouse, then FND-labelled pcMSCs were injected through the portal vein. **b** Bodistribution of FND-labelled pcMSCs in healthy kidney mouse model. Experiments were repeated in triplicate and error bars represent the standard deviation of the measurements. **c** Biodistribution of FND-labelled pcMSCs in ischemia–reperfusion kidney injury mouse model. Experiments were repeated in triplicate and error bars represent the standard deviation of uncertainty. Data are presented as mean ± standard deviation. Data were analyzed using Student’s *t*-test. *P value of < 0.05. **P value of < 0.01. ***P value of < 0.001. ****P value of < 0.0001

As it has been reported that MSCs will migrate to injured sites [266], we induced an ischemia–reperfusion injury to the left kidney in our animal model (Fig. 6a) and examined whether FND-labelled pcMSCs injected into the portal vein would appear in the injured kidney, to test the concept that MSCs will migrate to sites of injury. In our mouse model with healthy kidneys, the number of pcMSCs in the kidneys decreased over time (Fig. 6b, upper panel) and the decrease was evident in both the left and right kidneys. (Fig. 6b, lower panel). In contrast, in the mouse model with the injury the number of FND-labelled pcMSCs in the injured kidney was highest on day 5 (3%; Fig. 6c). As seen in the lower panel of Fig. 6c, the injured kidney (L kidney) had significantly more FND-labelled pcMSCs than the healthy kidney (R kidney). The percent of FND-labelled pcMSCs remained consistent over time (~ 0.25%) in the healthy right kidney (R kidney) (*P < 0.5, **P < 0.01, ***P < 0.001, ****P < 0.0001.) (Fig. 6c, lower panel). Given these data, it appears that the percentage of MSCs that migrate to kidneys is limited to about 4%, and it appears that the kidneys have the ability to redistribute MSCs in vivo. In addition to providing fast and accurate results, this technique is completely safe to the cell tissue. The FND-labelling technique does not alter any properties of the cell, including cell viability, proliferation, differentiation and immunomodulation, making this method very biocompatible.

### Clinical applications of MSCs in cell therapy: safety and potency

The potential and promise of MSC therapy is highly anticipated in recent and coming decades. As with all emerging new medical technologies, patient safety is always the first priority. As we have discussed, although the ability to modulate immune environment and promote tissue regeneration have been well reported in preclinical studies, the aspect regarding tumor induction or promotion is still one of the many concerns. The MSCs derived from different tissue origins or expanded under different culture conditions present different immune profiles which may result in tumor promotion [126]. Additionally, as the double sided blades of the MSCs’ strong immune modulation ability [262], evaluation of both the specific MSC properties as well as the patient’s immune conditions is strongly needed. The patient’s immune condition both before, during, and after treatment should be closely monitored.

Some reports showed that artificial engineering process may decrease the tumor induction and increase tumor-suppressing function of MSCs [263]. However, genetically engineered MSCs also raise other safety concerns. Although several clinical trials claimed the safety of MSC-treated patients, however, most of the trials only showed short-term safety and are without the examination of tumor-associated biomarkers [267, 268].

A recent systematic review and meta-analysis reappraised 55 randomized controlled trials and over 2000 patients to investigate the safety of systemically inoculated MSCs [39]. The risk of fever was significantly greater in the group of patients receiving MSCs. There was no significant increase in the risk of infection, thrombo-embolic events, malignancy or ectopic tissue formation, while the risk of death was significantly lower in the MSC-treated patients. Among the included studies, severe adverse events, including treatment related fever, in-stent thrombosis with death, acute coronary artery occlusions after intra-coronary delivery, grade 1 anaphlyactoid reaction, gastric ulcer perforation, hypersensitivity reaction, and anal cancer, have been reported to be possibly related to MSC treatment. Although the conclusion of the meta-analysis ends on a promising note, it was also emphasized that an a priori plan to monitor safety should be outlined in every clinical study design, including immediate allergic reactions, local complications (hematoma formation, local infection), vascular obstructions (dyspnea, oliguria, myocardial infarction, venous thromboembolic events), systemic complications (systemic infection, abnormal liver or renal function), malignancy or ectopic manifestation of implanted MSCs, and other disease-specific safety considerations [39].

Additionally, patients with medical history of ischemic diseases, cardiovascular diseases, lung fibrosis, concurrent neoplasm, and family history of hereditary cancer should be carefully reviewed during MSC treatment. The cell dose, infusion route and rate should be documented. The product profiles of the MSCs from different tissues and different generation processes, such as transcriptome, epigenome, proteomic data, cell populations, potential potency biomarkers, preclinical data from cell and animal studies, should be provided.

The therapeutic efficacy of MSCs in different disease indication is still under evaluation, as most of the studies to date have been limited to phase 1 and phase 2 studies (Fig. 1b and 1c, Additional file 2). As we have discussed in this review, the differences in MSC tissue origins and the variety of cell culture conditions would be some of the important factors determining MSC potency in vivo [269]. Thus, the development of surrogate potency assays using preclinical animal model is needed [270]. Recently the International Society for Cellular Therapy (ISCT) have announced some strategies to identify the potential effective factors of MSC action mechanism, including the combined the matrix assay and multiple techniques, such as quantitative RNA analysis for the specific genes, flow cytometry analysis for cell surface markers, and the protein-based assay of secretome [271]. Potency assessments in evaluating cell pharmacology, cell delivery route, as well as the cell-drug interaction are still under development to improve the MSC precision therapy [272–275]. Although the matrix assays were reported to serve as a platform to identify the biomarkers for MSC potency in vitro [276, 277], whether this in vitro assays are able to identify the MSC potency are still under discussion. For example, the use of allogeneic human peripheral blood mononuclear cells for mixed lymphocyte reaction (MLR) assays is a popular assay to demonstrate the MSC immunomodulation capacity. However, the lack of robustness, accuracy, and reproducibility is of concern [278–280]. Additionally, the correlation between the in vitro assays and in vivo pre-clinical/clinical data requires further evaluation.

Cryopreservation could be another factor affecting MSC potency. It has been documented that the MSC cryostorge, the so-called “cryo stun effect”, may decrease MSC therapeutic efficacy, leading to failures in MSC clinical trials [278]. Recently, a systematic review regarding the impact of cryopreservation on BMMSCs showed that the cryopreservation appears to affect the cell viability, apoptosis, cellular attachment, immunomodulation, and metabolism of BMMSCs [279]. Furthermore, these impaired viability or functions of the MSCs can be restored, partially or totally, by following an acclimation period [279–281], or by IFNγ licensing before cryopreservation [282].

In summary, the use of standardized potency assays should be incorporated into future MSC product release criteria. Thus, development of surrogate potency assays for different disease indications should be highlighted. The optimal process of cryopreservation and thawing may be another important factor requiring further attention.

## Conclusions

MSCs are a major cornerstone to the advancement of cell therapy, yet much remains to be learned about their pharmacokinetics and pharmacodynamics after systemic application in vivo. The different tissue origins of MSCs not only confer different biological activities that affect their therapeutic usefulness, but also raise the concern of different safety profiles. Many methods, including herein discussed fluorescent nanodiamond, are available for tracking inoculated MSCs in vivo, each with different advantages and disadvantages. These imaging platforms will facilitate future studies to discern and optimize the use of different MSCs for future clinical therapies.

## Supplementary Information

**Additional file 1. **Flowchart of MSC clinical study inclusion. As of October 11, 2020, 1,242 registered studies were identified on clinicaltrials.gov by searching keywords “mesenchymal stem cell” or “mesenchymal stromal cell”. After excluding studies with no longer available/ suspended/ temporarily not available/ terminated/ unknown/ withdrawn status, unknown phase information, and studies that did not use MSCs in their intervention arm, 639 studies remained. Nine of these 639 studies investigated MSCs from two tissue origins, generating a total of 648 studies for analysis.

**Additional file 2. **Breakdown of MSC-related clinical studies by disease indication. The included studies for analysis are as illustrated in Additional file 1.

## Data Availability

All relevant data are included in this published article.

## Abbreviations

AC-LCSC: Adenocarcinomas-lung cancer stem cells
AFP: Alpha-fetoprotein
ALDH: Aldehyde dehydrogenase
ALP: Alkaline phosphatase
Ang-1: Angiopoietin-1
APL: Acute promyelocytic leukemia
ATG5: Autophagy related 5
ATG7: Autophagy related 7
BAX: Bcl-2-associated X
Bcl-2: B-cell lymphoma 2
Bcl-xL: B-cell lymphoma-extra large
BCRP: Breast cancer resistance protein
BMP: Bone morphogenetic protein
CA-125: Cancer antigen 125
CAFs: Cancer-associated fibroblasts
CCL2: C–C motif chemokine 2 (MCP-1)
CCL5: C–C motif chemokine 5
CCR1: C–C chemokine receptor type 1
CD: Cytosine deaminase
CL: Cell lysate
CM: Conditioned medium
CSC: Cancer stem cells
CTL: Cytotoxic T-cell
CXCL1: C-X-C Motif Chemokine Ligand 1 (GRO-a)
CXCL8: C-X-C Motif Chemokine Ligand 8 (IL8)
CXCL12: C-X-C Motif Chemokine Ligand 12 (SDF-1)
CXCR1: C-X-C chemokine receptor type 1
CXCR2: C-X-C chemokine receptor type 2
CXCR4: C-X-C chemokine receptor type 4
CXCR7: C-X-C chemokine receptor type 7
CXCR12: C-X-C chemokine receptor type 12
DIABLO: Direct IAP-Binding protein with Low PI
ECM: Extracellular matrix
EGFR: Epidermal growth factor receptor
EMT: Epithelial–mesenchymal transition
EP: Prostaglandin E2 receptor
ERK: Extracellular signal–regulated kinase
ET1: Endothelin-1
EVs: Extracellular vesicles
Exos: Exosomes
FAK: Focal adhesion kinase
FND: Fluorescent nanodiamond
FoxO3a: Forkhead box class O 3a
GBM: Glioblastoma Multiforme
Glut-1: Glucose transporter type 1
GRO-a: Growth-regulated oncogene-alpha (CXCL1)
GSK3β: Glycogen synthase kinase 3β
hCG: Human chorionic gonadotropin
HDGF: Hepatoma-derived growth factor
HGF: Hepatocyte growth factor
HIF-1α: Hypoxia-inducible factor 1alpha
HK-2: Hexokinase-2
IBMIR: Instant blood-mediated inflammatory reaction
IDO: Indoleamine-2,3-dioxygenase
IGF: Insulin-like growth factor
IL1β: Interleukin 1 beta
IL6: Interleukin 6
IL8: Interleukin 8
IFN: Interferon
JAK: Janus kinase
JNK: C-Jun N-terminal kinase
LDH: Lactic dehydrogenase
Mad1: Mitotic arrest deficient 1
MAPK: Mitogen-activated protein kinase
MARCKS: Myristoylated Alanine Rich Protein Kinase C Substrate
MCP-1: Monocyte chemoattractant protein-1 (CCL2)
MDSC: Myeloid-derived suppressor cells
MMF: Magnetic modulation fluorescence
MMP: Matrix metalloproteinase
MSC: Mesenchymal stem/stromal cell
MWCO: Molecular weight cut off
NRG1: Neuregulin 1
NF-kB: Nuclear factor kappa-light-chain-enhancer of activated B cells
NK: Natural killer
OSM: Oncostatin M
PAI-1: Plasminogen activator inhibitor-1
PCNA: Proliferating cell nuclear antigen
PDCD4: Programmed cell death 4
PDGF: Platelet-derived growth factor
PDGFR: Platelet-derived growth factor receptor
PD-L1: Programmed death-ligand 1
PI3K: Phosphoinositide 3-kinase
PD: Pharmacodynamics
PK: Pharmacokinetics
PKM-2: Pyruvate Kinase M2
Plau/uPA: Urokinase-type plasminogen activator
pRb: Phosphorylated retinoblastoma protein
PTBP1: Polypyrimidine tract-binding protein 1
PTEN: Phosphatase and tensin homolog
RECK: Reversion-inducing-cysteine-rich protein with kazal motifs
SCC: Squamous cell carcinomas
SASP: Senescence-associated secretory phenotype
SDF-1: Stromal cell-derived factor-1(CXCL12)
Sema-7A: Semaphorin-7A
Smac: Second mitochondria-derived activator of caspases
STAT: Signal transducer and activator of transcription
TAM: Tumor-associated macrophage
TF: Tissue factor
TGF-β: Transforming growth factor beta
Th1: T-helper 1
TIMP: Tissue inhibitors of metalloproteinase
TMZ: Temozolomide
TNF-α: Tumor Necrosis Factor-α
TNFSF14: Tumor necrosis factor superfamily member 14
TRAIL: Tumor necrosis factor-related apoptosis-inducing ligand
VEGF: Vascular endothelial growth factor
XIAP: X-linked inhibitor of apoptosis protein
ZEB1: Zinc finger E-box binding homeobox 1
