# Supplementary material for: Mesenchymal stem/stromal cell-based therapy: mechanism, systemic safety and biodistribution for precision clinical applications
Source: J Biomed Sci. 2021 Apr 14;28:28. doi: 10.1186/s12929-021-00725-7 (PMC8043779; doi:10.1186/s12929-021-00725-7)
Supplement: Supplementary file 1 — Additional file 1. Flowchart of MSC clinical study inclusion. As of October 11, 2020, 1,242 registered studies were identified on clinicaltrials.gov by searching keywords “mesenchymal stem cell” or “mesenchymal stromal cell”. After excluding studies with no longer available/ suspended/ temporarily not available/ terminated/ unknown/ withdrawn status, unknown phase information, and studies that did not use MSCs in their intervention arm, 639 studies remained. Nine of these 639 studies investigated MSCs from two tissue origins, generating a total of 648 studies for analysis. [file 12929_2021_725_MOESM1_ESM.pdf]

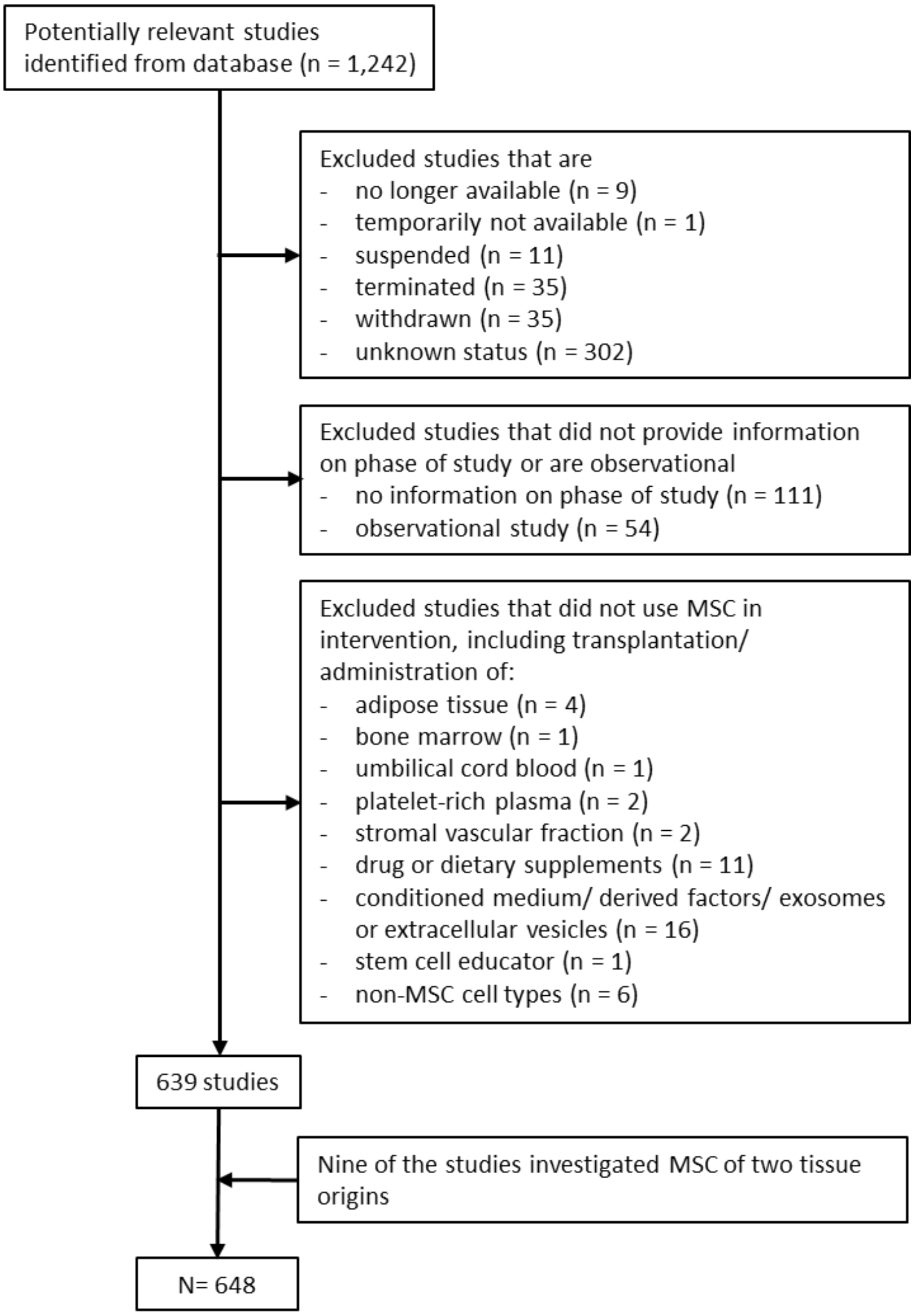

Supplementary Figure 1. Flowchart of MSC clinical study inclusion. As of October 11, 2020, 1,242 registered studies were identified on clinicaltrials.gov by searching keywords "mesenchymal stem cell" or "mesenchymal stromal cell". After excluding studies with no longer available/ suspended/ temporarily not available/ terminated/ unknown/ withdrawn status, unknown phase information, and studies that did not use MSCs in their intervention arm, 639 studies remained. Nine of these 639 studies investigated MSCs from two tissue origins, generating a total of 648 studies for analysis.
