# Supplementary material for: Mesenchymal stem/stromal cell-based therapy: mechanism, systemic safety and biodistribution for precision clinical applications
Source: J Biomed Sci. 2021 Apr 14;28:28. doi: 10.1186/s12929-021-00725-7 (PMC8043779; doi:10.1186/s12929-021-00725-7)
Supplement: Supplementary file 2 — Additional file 2. Breakdown of MSC-related clinical studies by disease indication. The included studies for analysis are as illustrated in Additional file 1. [file 12929_2021_725_MOESM2_ESM.pdf]

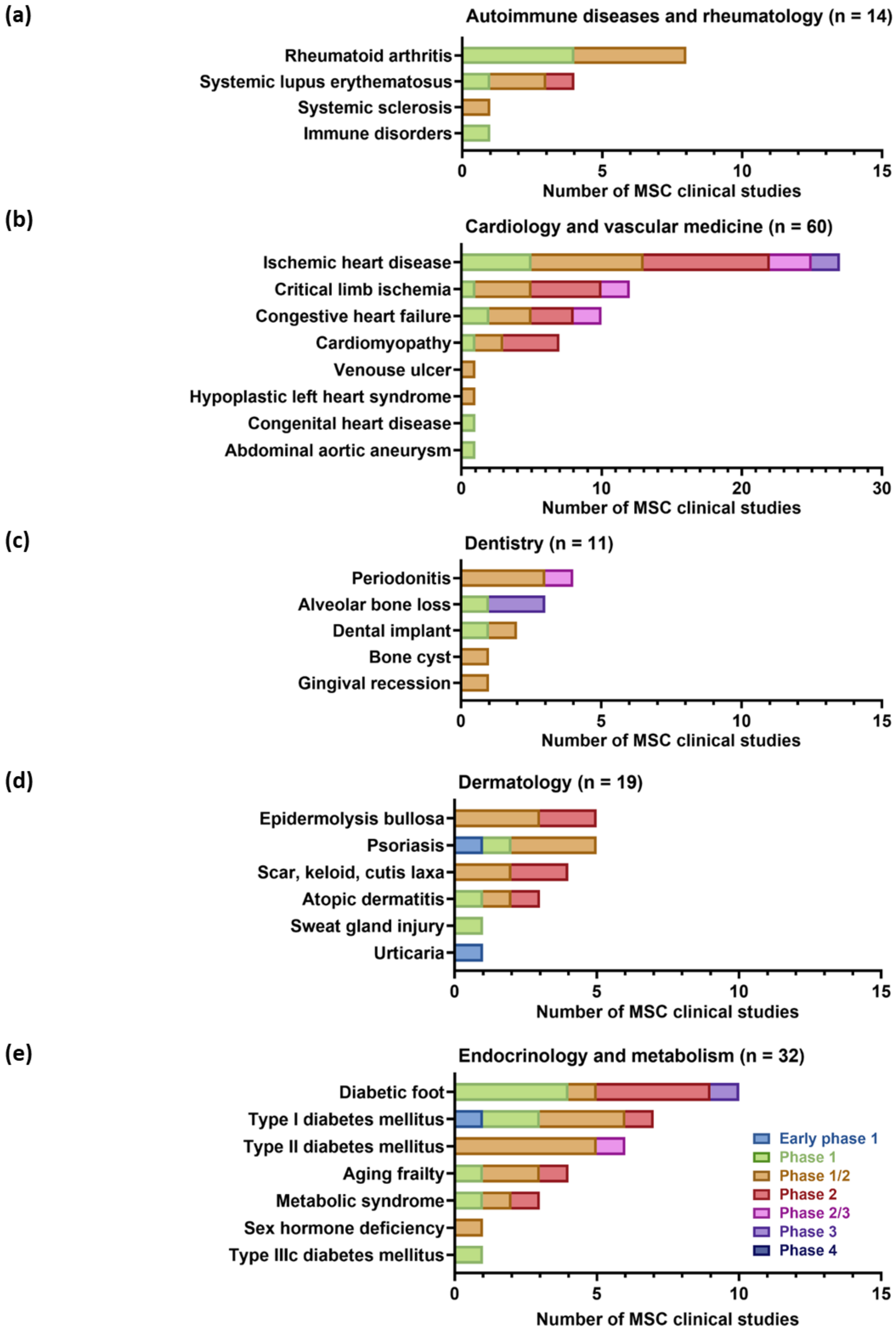

(a)
Autoimmune diseases and rheumatology (n = 14)
Rheumatoid arthritis
Systemic lupus erythematosus
Systemic sclerosis
Immune disorders
0
5
10
15
Number of MSC clinical studies
(b)
Cardiology and vascular medicine (n = 60)
Ischemic heart disease
Critical limb ischemia
Congestive heart failure
Cardiomyopathy
Venouse ulcer
Hypoplastic left heart syndrome
Congenital heart disease
Abdominal aortic aneurysm
0
10
20
30
Number of MSC clinical studies
(c)
Dentistry (n = 11)
Periodonitis
Alveolar bone loss
Dental implant
Bone cyst
Gingival recession
0
5
10
15
Number of MSC clinical studies
(d)
Dermatology (n = 19)
Epidermolysis bullosa
Psoriasis
Scar, keloid, cutis laxa
Atopic dermatitis
Sweat gland injury
Urticaria
0
5
10
15
Number of MSC clinical studies
(e)
Endocrinology and metabolism (n = 32)
Diabetic foot
Type I diabetes mellitus
Type II diabetes mellitus
Aging frailty
Metabolic syndrome
Sex hormone deficiency
Type IIIc diabetes mellitus
Early phase 1
Phase 1
Phase 1/2
Phase 2
Phase 2/3
Phase 3
Phase 4
0
5
10
15
Number of MSC clinical studies

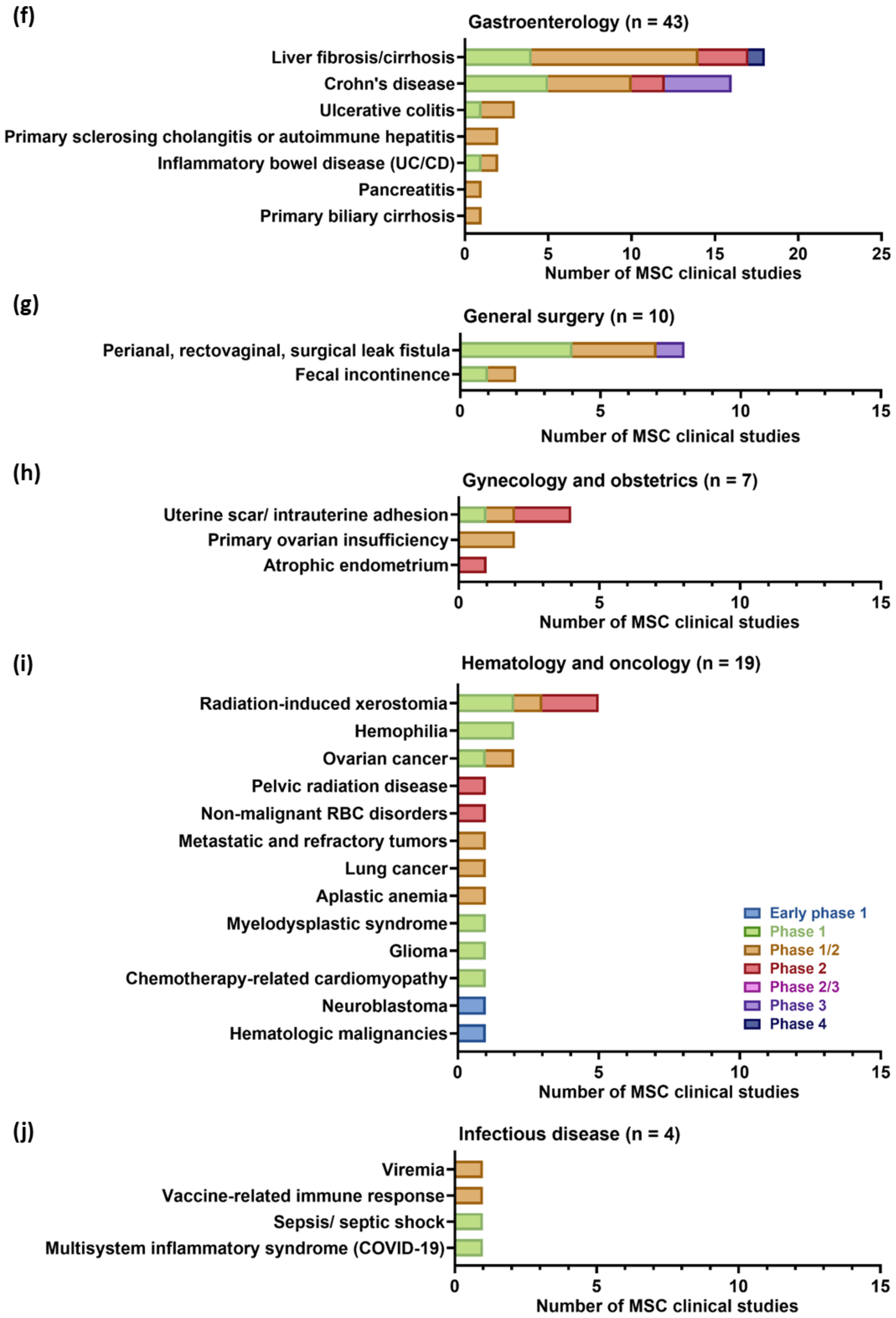
(f)
Gastroenterology (n = 43)
Liver fibrosis/cirrhosis
Crohn's disease
Ulcerative colitis
Primary sclerosing cholangitis or autoimmune hepatitis
Inflammatory bowel disease (UC/CD)
Pancreatitis
Primary biliary cirrhosis
0
5
10
15
20
25
Number of MSC clinical studies
(g)
General surgery (n = 10)
Perianal, rectovaginal, surgical leak fistula
Fecal incontinence
0
5
10
15
Number of MSC clinical studies
(h)
Gynecology and obstetrics (n = 7)
Uterine scar/ intrauterine adhesion
Primary ovarian insufficiency
Atrophic endometrium
0
5
10
15
Number of MSC clinical studies
(i)
Hematology and oncology (n = 19)
Radiation-induced xerostomia
Hemophilia
Ovarian cancer
Pelvic radiation disease
Non-malignant RBC disorders
Metastatic and refractory tumors
Lung cancer
Aplastic anemia
Myelodysplastic syndrome
Glioma
Chemotherapy-related cardiomyopathy
Neuroblastoma
Hematologic malignancies
Early phase 1
Phase 1
Phase 1/2
Phase 2
Phase 2/3
Phase 3
Phase 4
0
5
10
15
Number of MSC clinical studies
(j)
Infectious disease (n = 4)
Viremia
Vaccine-related immune response
Sepsis/ septic shock
Multisystem inflammatory syndrome (COVID-19)
0
5
10
15
Number of MSC clinical studies

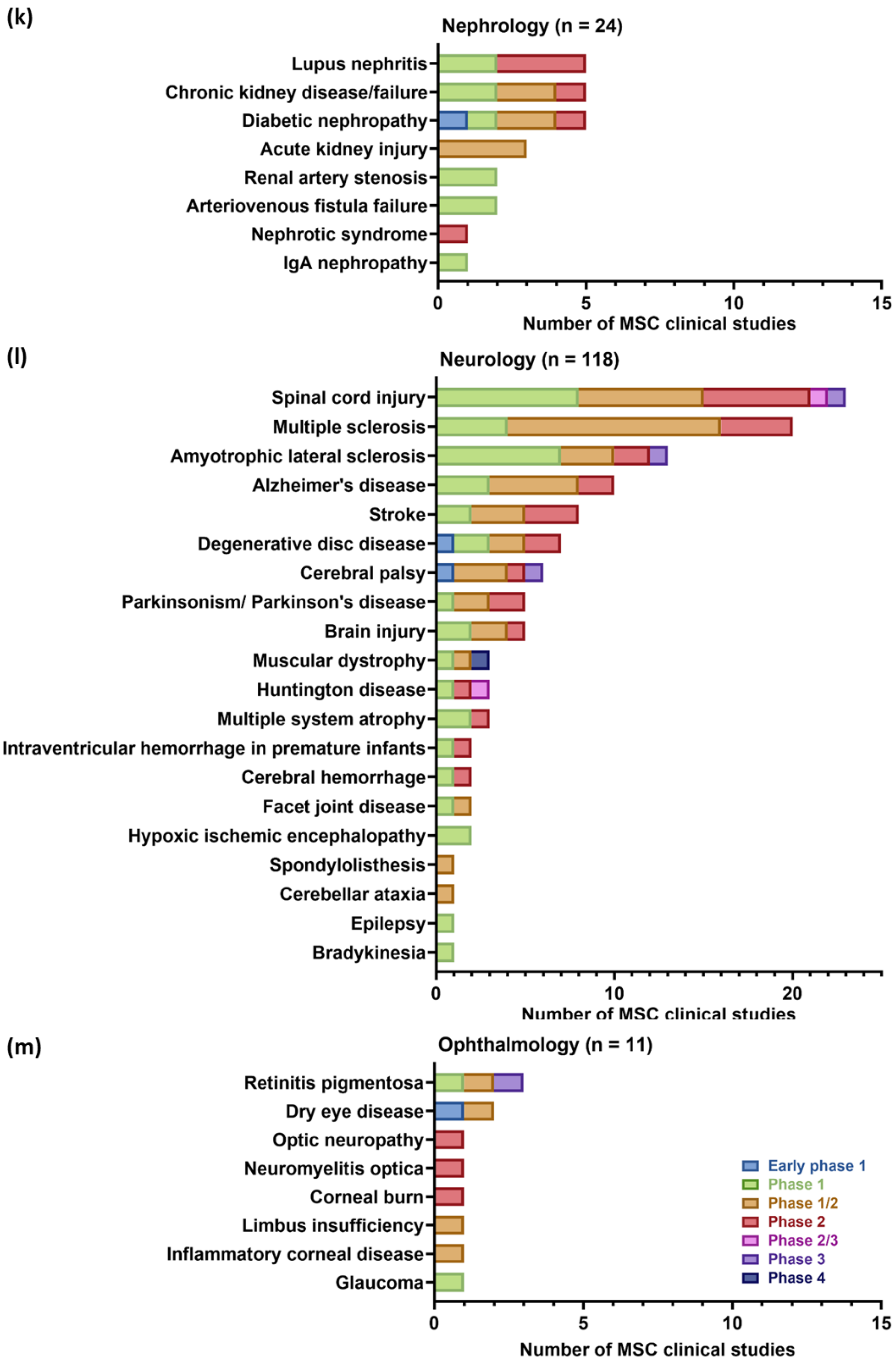

(k)
Nephrology (n = 24)
Lupus nephritis
Chronic kidney disease/failure
Diabetic nephropathy
Acute kidney injury
Renal artery stenosis
Arteriovenous fistula failure
Nephrotic syndrome
IgA nephropathy
0
5
10
15
Number of MSC clinical studies
(l)
Neurology (n = 118)
Spinal cord injury
Multiple sclerosis
Amyotrophic lateral sclerosis
Alzheimer's disease
Stroke
Degenerative disc disease
Cerebral palsy
Parkinsonism/ Parkinson's disease
Brain injury
Muscular dystrophy
Huntington disease
Multiple system atrophy
Intraventricular hemorrhage in premature infants
Cerebral hemorrhage
Facet joint disease
Hypoxic ischemic encephalopathy
Spondylolisthesis
Cerebellar ataxia
Epilepsy
Bradykinesia
0
10
20
Number of MSC clinical studies
(m)
Ophthalmology (n = 11)
Retinitis pigmentosa
Dry eye disease
Optic neuropathy
Neuromyelitis optica
Corneal burn
Limbus insufficiency
Inflammatory corneal disease
Glaucoma
0
5
10
15
Number of MSC clinical studies
Early phase 1
Phase 1
Phase 1/2
Phase 2
Phase 2/3
Phase 3
Phase 4

(n)

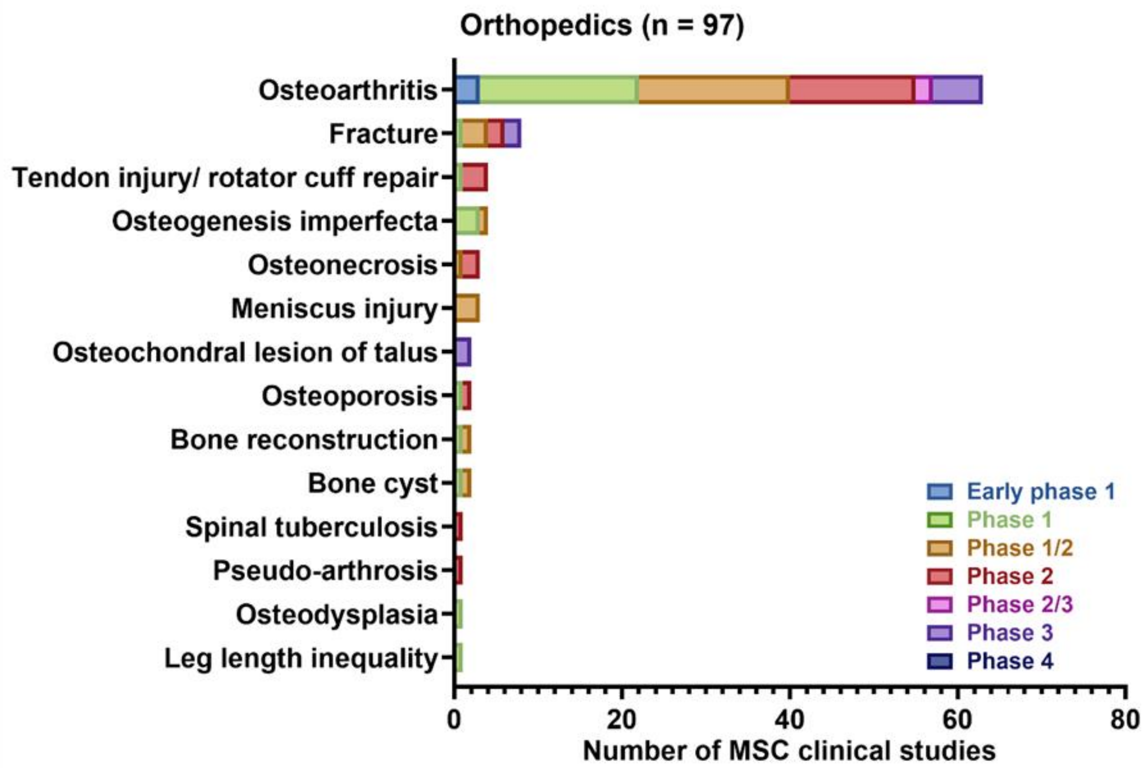
Orthopedics (n = 97)
Osteoarthritis
Fracture
Tendon injury/ rotator cuff repair
Osteogenesis imperfecta
Osteonecrosis
Meniscus injury
Osteochondral lesion of talus
Osteoporosis
Bone reconstruction
Bone cyst
Spinal tuberculosis
Pseudo-arthrosis
Osteodysplasia
Leg length inequality
Early phase 1
Phase 1
Phase 1/2
Phase 2
Phase 2/3
Phase 3
Phase 4
0
20
40
60
80
Number of MSC clinical studies

(o)

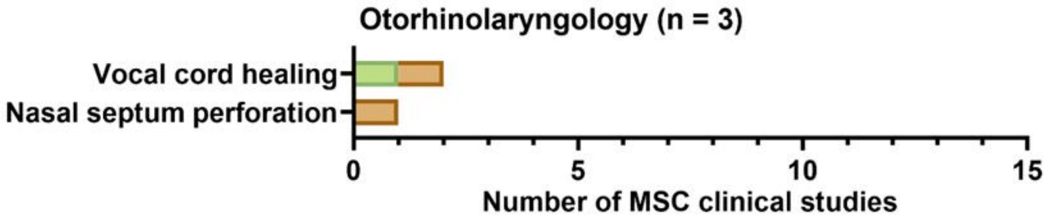
Otorhinolaryngology (n = 3)
Vocal cord healing
Nasal septum perforation
0
5
10
15
Number of MSC clinical studies

(p)

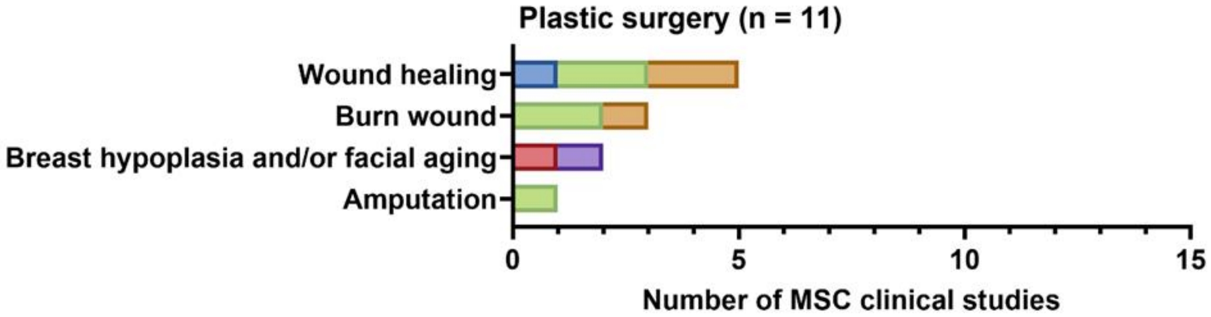
Plastic surgery (n = 11)
Wound healing
Burn wound
Breast hypoplasia and/or facial aging
Amputation
0
5
10
15
Number of MSC clinical studies

(q)

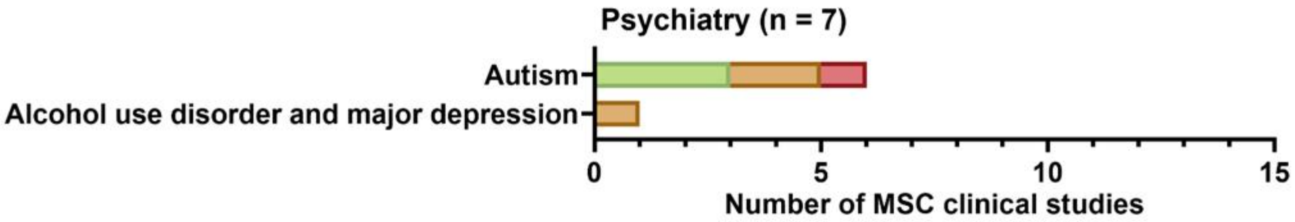
Psychiatry (n = 7)
Autism
Alcohol use disorder and major depression
0
5
10
15
Number of MSC clinical studies

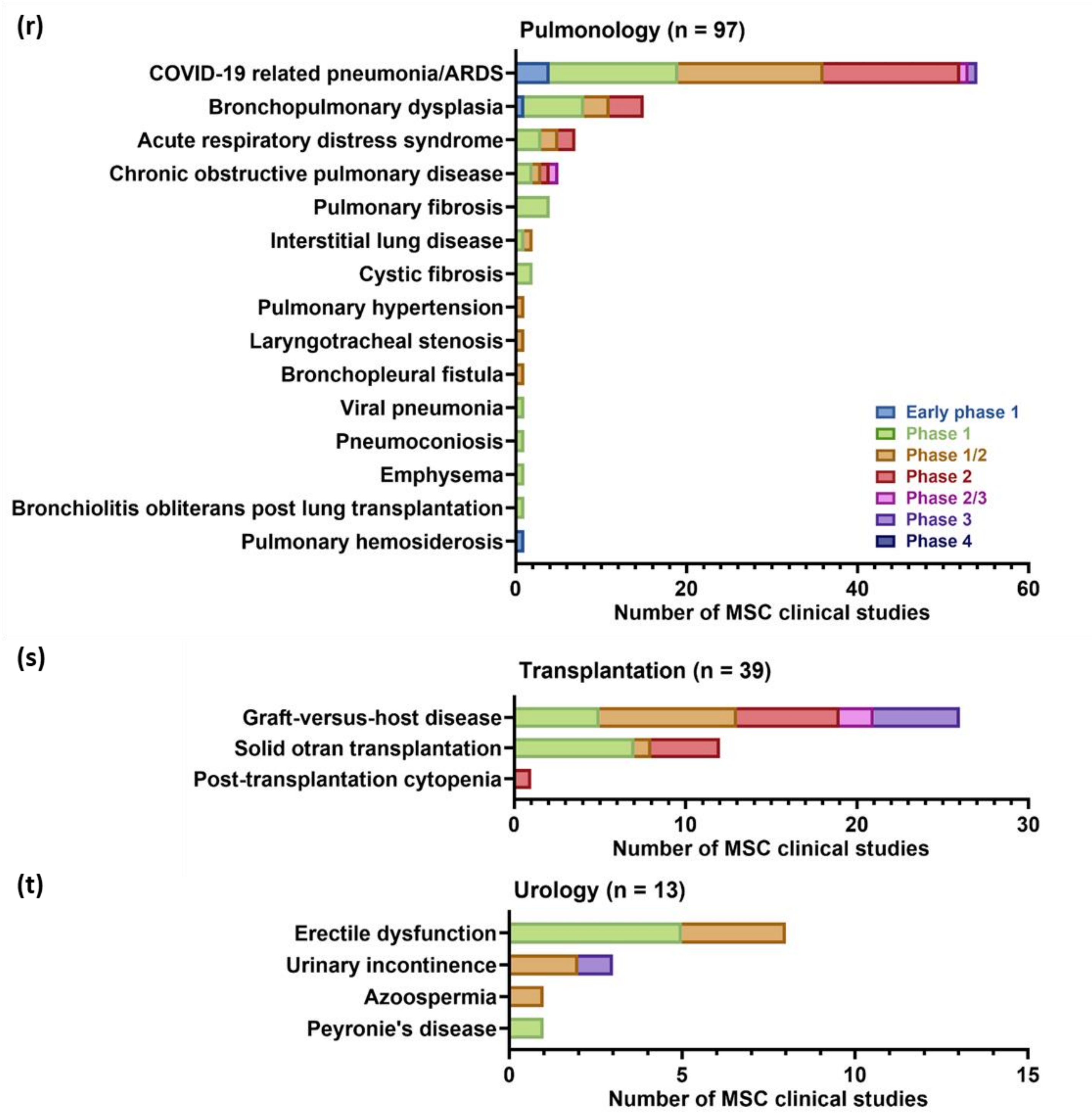

Supplementary Figure 2. Breakdown of MSC-related clinical studies by disease indication. The included studies for analysis are as illustrated in Additional file 1.
